# Exploring the transcriptome of resident spinal microglia after collagen antibody–induced arthritis

**DOI:** 10.1097/j.pain.0000000000001394

**Published:** 2018-12-28

**Authors:** Teresa Fernandez-Zafra, Tianle Gao, Alexandra Jurczak, Katalin Sandor, Zoe Hore, Nilesh M. Agalave, Jie Su, Johanna Estelius, Jon Lampa, Tomas Hokfelt, Zsuzsanna Wiesenfeld-Hallin, Xiaojun Xu, Franziska Denk, Camilla I. Svensson

**Affiliations:** a Department of Physiology and Pharmacology, Karolinska Institutet, Stockholm, Sweden; b Wolfson Centre for Age-Related Diseases, King's College London, London, United Kingdom; c Rheumatology Unit, Department of Medicine, Karolinska Institutet, Stockholm, Sweden; d Department of Neuroscience, Karolinska Institutet, Stockholm, Sweden

**Keywords:** Chronic pain, Arthritis, Microglia, Sex difference, Spinal cord, Minocycline, RNA-seq

## Abstract

Recent studies have suggested a sexually dimorphic role of spinal glial cells in the maintenance of mechanical hypersensitivity in rodent models of chronic pain. We have used the collagen antibody–induced arthritis (CAIA) mouse model to examine differences between males and females in the context of spinal regulation of arthritis-induced pain. We have focused on the late phase of this model when joint inflammation has resolved, but mechanical hypersensitivity persists. Although the intensity of substance P, calcitonin gene–related peptide, and galanin immunoreactivity in the spinal cord was not different from controls, the intensity of microglia (Iba-1) and astrocyte (glial fibrillary acidic protein) markers was elevated in both males and females. Intrathecal administration of the glial inhibitors minocycline and pentoxifylline reversed mechanical thresholds in male, but not in female mice. We isolated resident microglia from the lumbar dorsal horns and observed a significantly lower number of microglial cells in females by flow cytometry analysis. However, although genome-wide RNA sequencing results pointed to several transcriptional differences between male and female microglia, no convincing differences were identified between control and CAIA groups. Taken together, these findings suggest that there are subtle sex differences in microglial expression profiles independent of arthritis. Our experiments failed to identify the underlying mRNA correlates of microglial actions in the late phase of the CAIA model. It is likely that transcriptional changes are either subtle and highly localised and therefore difficult to identify with bulk isolation techniques or that other factors, such as changes in protein expression or epigenetic modifications, are at play.

## Abstract 2

Supplemental Digital Content is Available in the Text.

Glial inhibitors only reverse mechanical hypersensitivity in male mice subjected to arthritis. No obvious arthritis-related transcriptional difference was identified between male and female spinal microglia.

## 1. Introduction

Rheumatoid arthritis (RA) is an autoimmune disease that causes joint swelling and synovitis leading to progressive cartilage and bone destruction.^31^ Chronic pain is one of the most debilitating symptoms of this process^22,52^ and has long been attributed to peripheral inflammation. However, subgroups of patients report pain despite good medical control.^2,3^ Moreover, RA patients can develop a generalized increase in pain sensitivity at distant sites from the inflamed joint,^20,27,41^ suggesting an involvement of the central nervous system.^32^ This notion is supported by studies reporting elevated cytokine levels in the cerebrospinal fluid of patients and in animal models of RA.^25,26,35^

Microglia and astrocytes have emerged as crucial players in the maintenance of mechanical hypersensitivity,^1,6,14,17,35,56^ which on activation synthesize and release factors that facilitate neuronal excitability and transmission of nociception.^45,55^ Moreover, intrathecal delivery of glial inhibitors, such as minocycline or pentoxifylline, can prevent or reverse mechanical hypersensitivity in pain models.^15,23^

Although arthritis and autoimmunity research has been fairly balanced in the consideration of both sexes, most preclinical pain research has been performed in male animals. This is problematic in the context of studying pain in arthritis because RA, like other autoimmune diseases, is more likely to occur in women.^24^ Furthermore, in an experimental setting, many authors^5,33^—with some exceptions^43,44^—have come to the conclusion that there are sexual dimorphisms in how nociception and potentially other sensory stimuli^21^ are processed. Recently, an effort has been made to include female rodents when studying models of chronic pain. As it is usual when scientific explorations are at an early stage, the results as well as their accompanying potential explanations have been mixed. A few laboratories have suggested that mechanical allodynia is reversed only in male but not in female mice after intrathecal delivery of microglial inhibitors.^48,50^ The mechanisms underlying this phenomenon are still under debate.^29,48^ No clear sex differences have so far been reported in the context of pain in arthritis models.^1,35^

Here, we used the collagen antibody–induced arthritis (CAIA) model to investigate sexual dimorphisms in spinal signal transmission. In male mice, we have previously shown that spinal neuropeptide expression remains unchanged on CAIA induction. Instead, we obtained evidence for the contribution of microglia and astrocytes in arthritis-induced pain in male mice. For instance, we demonstrated changes in microglial activation during the late phase of the CAIA model, when mechanical hypersensitivity persists but joint inflammation has resolved.^1,6^ Moreover, we showed that intrathecal injection of drugs that interfere with astrocytic activity (pentoxifylline or c-Jun N-terminal kinase inhibitors) were able to reverse CAIA-induced mechanical hypersensitivity in male mice.^6^ In the current study, we set out to confirm these findings—this time in both male and female mice. In addition to behavioural and immunohistochemical analyses, we also performed genome-wide RNA-sequencing (RNA-seq) during the late phase of the CAIA model to further characterize the role of these cells in the context of arthritis-induced pain, and to identify targets that can be used for intervention. We hypothesized that microglia would play a similar role in females compared with males, given that it has been reported that this cell type is associated with pain-like behaviour in female rats subjected to collagen-induced arthritis.^14^ Interestingly, our results point towards a more complicated picture.

## 2. Materials and methods

### 2.1. Animals

Experimental procedures were performed under ethical approval by the Northern Stockholm Animal Research Committee. Adult male and female Balb/cAnNRj (Janvier Labs, Le Genest-Saint-Isle, France), CBA (Harlan) (12-18 weeks old), and C57BL/6JRj (Janvier Labs) mice were used and housed in groups (3-5 mice per cage) in a temperature- and humidity-controlled environment with a 12-hour light/dark cycle and access to food and water ad libitum. Whole spinal cord experiments were performed at King's College London on 10-week old-naive male and female C57BL/6J mice in accordance with UK Home Office Legislation (Scientific Procedures Act 1986).

### 2.2. Collagen antibody–induced arthritis

For induction of arthritis, mice were injected intravenously (1.25-1.5 mg) with an anticollagen type-II cocktail containing 5 different monoclonal autoantibodies (Chondrex, Redmond, WA) at day 0 followed by an intraperitoneal injection of lipopolysaccharide (25 μg in 100 μL; serotype 0111:B4; Chondrex, or 35 μg in 100 μL; serotype 055:B5; Sigma) at day 5.

### 2.3. Scoring of arthritis

Joint inflammation of the fore and hind paws was evaluated visually as previously described.^6^ Briefly, 1 point was given for every inflamed toe or knuckle. Each paw, ankle, or wrist was awarded 2.5 or 5 points if moderately or severely inflamed, respectively, resulting in a maximum arthritis score of 15 points per leg and 60 points per mouse. Animals that did not reach a minimum of 12 points in the hind paws at the peak of inflammation were excluded from the study.

### 2.4. Mechanical hypersensitivity

Before testing, mice were acclimatized for 30 to 40 minutes in individual Plexiglas compartments with a wire mesh bottom. Mechanical hypersensitivity was assessed by applying calibrated Von Frey filaments (Optihair) of incremental force (0.03–3.30*g*) to the plantar surface of the hind paw using the up–down method.^10^ The 50% paw withdrawal threshold (force at which the animal reacts 50% of the time) was calculated in grams for both hind paws and averaged. Three baseline testing sessions were performed on separate days and the average calculated for each animal. Results are presented as the average 50% withdrawal threshold for each group.

### 2.5. Intrathecal injections

Female and male mice were injected intrathecally with minocycline (30 µg in 5 µL, M9511; Sigma, Neustadt an der Weinstraße, Germany), pentoxifylline (30 µg in 5 µL, P1784; Sigma), or saline (5 µL, vehicle control) under light isoflurane anaesthesia in the late phase (>60 days after CAIA induction).

### 2.6. Immunohistochemistry

At 15 and 54 days after anticollagen type-II antibody cocktail or saline injection, male and female mice were deeply anaesthetized and transcardially perfused with saline (0.9% NaCl) followed by a fixative solution (4% paraformaldehyde with 0.2% picric acid in 0.16 M phosphate buffer). Lumbar spinal cords were dissected, postfixed for 90 minutes at 4°C, and cryoprotected with 10% sucrose in 0.1 M phosphate buffer for 48 hours at 4°C. Spinal cords were then embedded in OCT compound (Tissue-Tek) and cut at 20 μm with a cryostat (Microm). Tissue sections were incubated overnight with primary antibodies against calcitonin gene–related peptide (CGRP) (1:32,000^38^), substance P (SP) (1:4000^13^), galanin (1:4000^53^), glial fibrillary acidic protein (GFAP) (1:8000, Dako), and ionized calcium-binding adapter molecule 1 (IBA-1) (1:2000, Wako) at 4°C. Immunoreactivity was visualized with the TSA Plus kit (Perkin Elmer, Waltham, MA) as previously described.^8^ Images were captured with a LSM710 confocal laser scanning microscope (Carl Zeiss, Oberkochen, Germany), and the integrated signal intensity was measured after background subtraction in 3 sections per animal using Image J (NIH). Results are shown as the percentage change in signal intensity in CAIA compared with the saline group.

### 2.7. Microglial isolation

Male and female mice (naive, saline injected, or subjected to CAIA) were sacrificed by anesthetic overdose and transcardially perfused with ice-cold Hank's balanced salt solution (HBSS). Spinal cords were flushed out using a HBSS-filled syringe inserted in the tail end of the spinal column. Lumbar dorsal horns were dissected, and dounce homogenized in fluorescence-activated cell sorting (FACS) buffer (0.4% bovine serum albumin; 15-mM HEPES; and 2-mM EDTA in HBSS) into a cell suspension that was centrifuged (430*g* for 30 minutes at 17°C) over a 37%/70% Percoll gradient (Sigma). Microglial cells were isolated from the interface and the total cell number determined using a haemocytometer. For each experiment, the lumbar dorsal horns of 2 mice were pooled into one Percoll gradient, constituting one n per sample group (2 × saline/naive and 2 × CAIA).

### 2.8. FACS and qRT-PCR of whole naive spinal cord

Naive male and female C57BL6/J mice (Harlan, 8 weeks old) were PBS perfused, and microglia were extracted in batches of 4 (n = 2, male and female) and stained for flow cytometry as described above. Fluorescence-activated cell sorting was performed using a BD FACS Aria II Cell Sorter at the National Institute for Health Research Biomedical Research Centre flow core facility at King's College London. Live, single CD45/CD11b double positive cells were collected into RLT buffer for later batch-controlled RNA extraction with a QIAGEN RNeasy Micro Kit (74,004) as described above (Qiagen, Venlo, The Netherlands). The RNA obtained was of high quality ranging from 75 to 1490 pg/µL (average 451 pg/µL) with RIN values of 8.2 to 10 (average 9). The material was amplified using the Smart-seq2 protocol,^39,40^ according to the instructions provided by the authors. Quantitative reverse transcriptase polymerase chain reaction was conducted using primer pairs listed in Table 1, after having confirmed their efficiency and specificity (through melt curve and agarose gel analysis). In a separate experiment, additional qRT-PCR was performed on the lumbar part of spinal cords from naive male and female (n = 11-13/group) C57BL6/J and Balb/cAnNRj mice (Janvier, 12-18 weeks old). Flash frozen tissue was homogenized with bead Tissue Lyser (Qiagen) in Trizol (Thermo Fisher Scientific, Waltham, MA), and mRNA was extracted according to the manufacturer's protocol. The cDNA was prepared by reverse transcription and subjected to qRT-PCR with ABI 7900HT system, using TaqMan probes (Applied Biosystems, Foster City, CA): *Iba-1* (*Mm00479862_g1), Cd11b (Mm00434455_m1),* and *GAPDH (Mm99999915_g1).* Threshold cycle values in each sample were used to calculate the number of cell equivalents in the test samples using the standard curve method.^7^ The data were normalized to GAPDH mRNA levels and expressed as relative expression units.

**Table 1:**
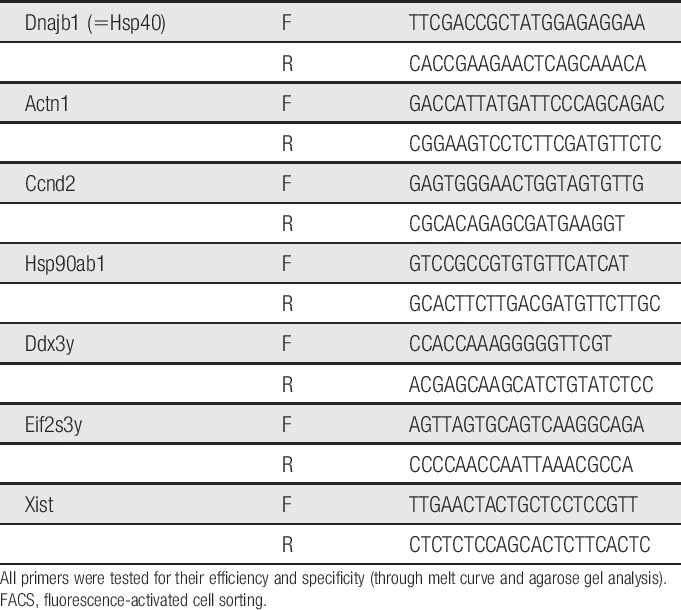
Primers used for qRT-PCR of FACS-purified mouse spinal cord microglia.

### 2.9. FACS of lumbar dorsal horns from CAIA and control mice

Microglial cells were centrifuged (230*g* for 3 minutes at 4°C) and stained with a monoclonal rat anti-Fcrls antibody (1:300; clone 4G11, kindly donated by Dr. Oleg Butovsky, Harvard, MA) for 20 minutes on ice. After a wash with FACS buffer and a second spin, cells were incubated for 20 minutes on ice with a goat anti-rat IgG secondary antibody conjugated to APC (1:400, clone Poly4054; Biolegend, San Diego, CA). Cells were washed and spun again, followed by staining with directly conjugated rat anti-mouse CD45-PE (1:400, clone 30-F11; Biolegend) and rat anti-mouse CD11b-FITC (1:400, clone 3A33; Abcam) antibodies for 20 minutes on ice. After washing, spinning, and staining dead cells with Sytox blue (1:1500; Life Technologies, Carlsbad, CA), cells were sorted with a BD Influx flow cytometer (core facility at the Center for Molecular Medicine, Karolinska Institutet), and purified microglia were collected and lysed in RLT buffer (Qiagen, 79,216) containing 1% β-mercaptoethanol. Unstained cells and single staining controls (Sytox blue stained dead cells and BD Comp beads [BD Biosciences] for fluorescein isothiocyanate, phycoerythrin, and allophycocyanin fluorochromes) were used for compensation. To aid the gating, we used a fluorescence-minus-one control, where the Fcrls primary antibody was omitted to control for unspecific fluorescence of the APC secondary antibody. Naive and CAIA-injected animals in the inflammatory phase (day 9 or day 17) were stained for all 3 markers (Fcrls, CD45, and CD11b), whereas saline- and CAIA-injected animals in the postinflammatory phase (days 55-65) were only stained for CD45 and CD11b. For data analysis in FlowJo, gates were kept constant across conditions in the same experimental set (saline/naive vs CAIA). On average, 3200 microglia were sorted for these experiments in group-matched batches. Only 3 of 31 samples contained less than 1000 cells.

### 2.10. RNA extraction

RNA from lumbar dorsal horn–sorted microglial cells was extracted using a QIAGEN RNeasy Micro Kit (74,004) according to the manufacturer's instructions with minor modifications. If sorted samples contained less than 2000 cells, then 2 samples from the same condition were pooled into one, constituting one n per sample group (2-4 × saline and 2-4 × CAIA mice). RNA quantity and integrity were assessed with a Bioanalyzer using Pico Chips (Agilent Technologies, Santa Clara, CA).

### 2.11. Sequencing

cDNA library preparation and RNA sequencing were performed by the High Throughput Genomics Group at the Wellcome Trust Centre for Human Genetics (Oxford University). The cDNA libraries were prepared with a SMARTer Ultra Low Input RNA Kit (Clontech, Mountain View, CA). Samples were amplified and multiplexed on an Illumina HiSeq4000 platform to yield 75-bp fragments at a depth of at least 27 million reads per sample. Each sample sequenced (n = 23) contained lumbar dorsal horn–sorted microglia obtained from a pool of 2 to 4 saline or CAIA mice.

### 2.12. RNA-seq data analysis

Relevant quality control was performed using the RSeQC algorithm.^57^ In particular, we checked for gene body coverage because SMARTer library prep can result in significant 3′ bias. Reads were aligned to the mm10 mouse genome using STAR^18^ (default parameters plus: --outFilterMultimapNmax 1 --clip3pAdapterSeq “TGGTATCAACGCAGAGTAC”). Alignment rates were between 83% and 92% (89% on average). FPKM values were obtained using the cufflinks algorithm^54^ on the Galaxy Freiburg server. Genes with an fragments per kilobase million value of at least 1 in a majority of samples per group were considered expressed. For differential gene expression, we generated count data with the aid of the feature counts algorithm^28^ and fed them into the Deseq2 algorithm^30^ in R. Raw, and processed data are available under gene expression omnibus accession GSE108896.

### 2.13. Statistics

For data besides RNA-seq, differences between 2 groups were analysed with a 2-tailed unpaired student *t* test. Comparisons between more than 2 groups were performed with 1-way analysis of variance for 1 independent variable or 2-way analysis of variance for 2 independent variables. Multiple comparisons were adjusted with Bonferroni post hoc test. Data are shown as mean ± SEM.

## 3. Results

### 3.1. CAIA induces transient joint inflammation and persistent mechanical hypersensitivity in male and female mice

**Figure 1.**
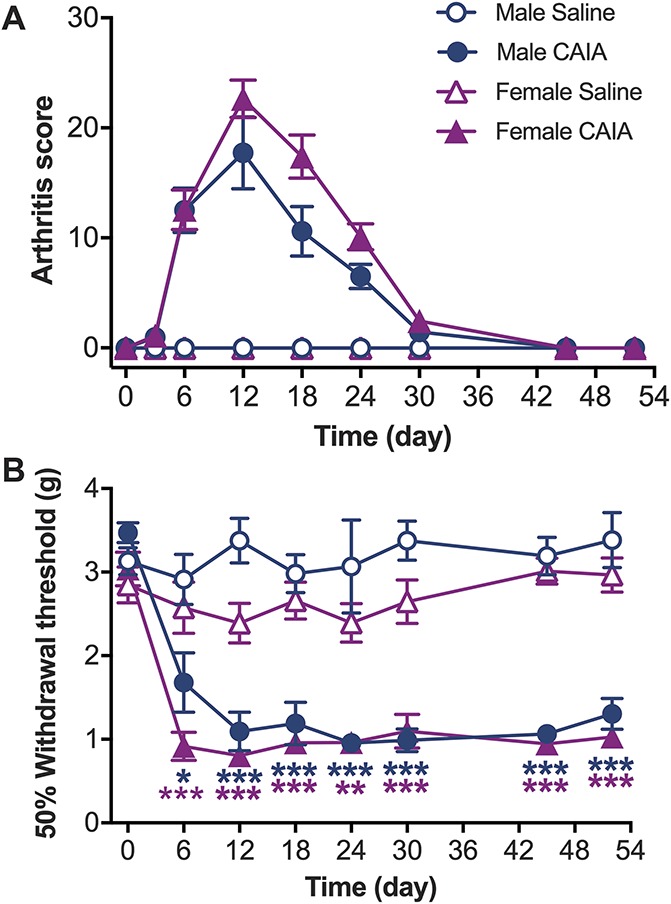
Collagen antibody–induced arthritis (CAIA) induces transient joint inflammation and persistent mechanical hypersensitivity in male and female mice. (A) Joint inflammation was assessed visually by counting the number of inflamed digits, paws, wrists, and ankles (arthritis score). Male and female CAIA groups developed transient joint inflammation, whereas saline injected controls did not show any signs of inflammation. (B) Mechanical hypersensitivity was assessed by measuring paw withdrawal thresholds using von Frey filaments. Both male and female CAIA groups developed persistent mechanical hypersensitivity while the saline injected groups did not. Data are presented as mean ± SEM, males n = 8; females n = 12 mice per group, CAIA vs saline, ***P > 0.001, **P > 0.01, *P > 0.05.

Adult male and female mice were subjected to CAIA by an intravenous (i.v.) injection of a collagen type-II antibody cocktail on day 0, followed by an intraperitoneal (i.p.) injection of LPS on day 5 to boost and synchronize the arthritogenic response. We found significant differences in CAIA incidence between males and females, whereas 90% (27/30) of females developed CAIA, only 53.5% (23/43) of males did. Male and female mice that developed CAIA presented visually apparent joint swelling and redness from day 6, reaching a peak in arthritis score around day 12 and gradually returning to baseline levels around day 30 (Fig. 1A). Although joint inflammation was transient in all mice, mechanical hypersensitivity, which was present from day 6, persisted up until the last testing point at day 52 (*P* < 0.05 to *P* < 0.001 on days 6-52 compared with saline controls) (Fig. 1B). We refer to the period characterized by joint swelling and mechanical hypersensitivity as the “inflammatory phase,” and the period where joint inflammation has resolved but mechanical hypersensitivity persists as the “postinflammatory phase” or “late phase.” Notably, saline-matched controls did not present any signs of inflammation or mechanical hypersensitivity at any time point.

### 3.2. CAIA does not alter expression levels of spinal CGRP, SP, or galanin

**Figure 2.**
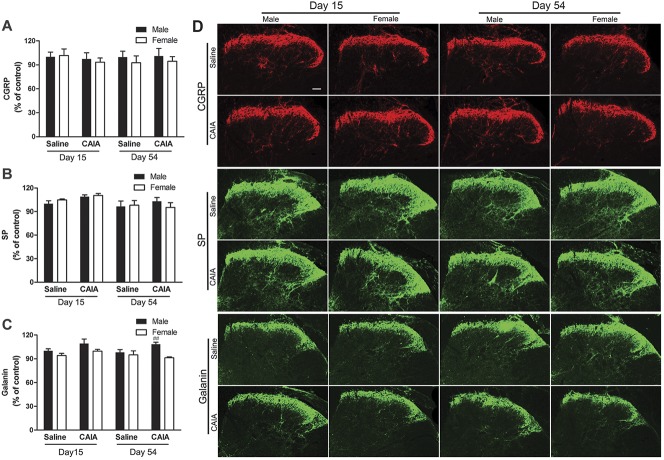
Collagen antibody–induced arthritis (CAIA) does not alter the expression levels of CGRP, SP or galanin in the spinal dorsal horn. Relative expression based on assessment of fluorescence intensity of (A) CGRP, (B) SP, and (C) galanin, as well as (D) representative images of immunoreactivity in the lumbar spinal cord of male and female mice 15 and 54 days after CAIA induction. Data are presented as mean ± SEM, n = 8 mice per group, male vs female CAIA mice on day 54, ^##^*P* > 0.01. CGRP, calcitonin gene–related peptide; SP, substance P.

Previously, we have shown that male mice subjected to CAIA do not display spinal changes in neuropeptides associated with pain (CGRP, SP, and galanin) during the inflammatory and postinflammatory phases of this model. To investigate whether female mice show altered expression of these neuropeptides after CAIA induction, we compared male and female lumbar spinal cords at the peak of the inflammatory phase (15 days) and during the postinflammatory phase (54 days). In line with our previous work, we found no change in expression levels for CGRP, SP, nor galanin in male mice at the time points examined after CAIA induction (Fig. 2). Nevertheless, comparing male and female mice in the late phase, we found that galanin expression levels were modestly but statistically higher in male compared with female CAIA mice in the late phase (Fig. 2C). However, there was no significant difference in spinal galanin levels between control and CAIA mice, independent on sex and time point. No changes in CGRP or SP expression were observed in female mice after CAIA induction nor in saline controls from both sexes.

### 3.3. CAIA leads to an increase in spinal GFAP and IBA-1 immunoreactivity in male and female mice

**Figure 3.**
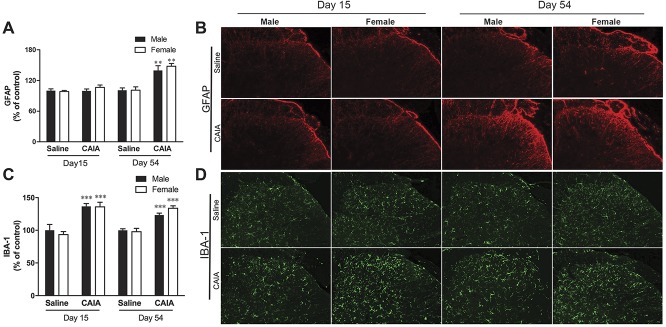
Collagen antibody–induced arthritis (CAIA) leads to an increase in spinal GFAP and IBA-1 immunoreactivity in male and female mice. Relative expression based on assessment of fluorescence intensity of (A) GFAP and (C) IBA-1 and (B and D) representative images of immunoreactivity in the lumbar spinal cord of male and female mice 15 and 54 days after CAIA induction. Significant increase of the astrocyte marker GFAP was observed only in the late phase of CAIA in both males and females, whereas increase in the signal intensity of the microglia marker IBA-1 was detected in both CAIA phases in both male and female mice. Data are presented as mean ± SEM, n = 8 mice per group, CAIA vs saline, ***P > 0.001, **P > 0.01. GFAP, glial fibrillary acidic protein.

We have previously shown that male mice subjected to CAIA display signs of microglial activation both in the inflammatory and late phases, whereas they only show increased levels of GFAP in the postinflammatory phase of the model.^1,6^ To understand whether female mice show the same pattern of glial reactivity, we stained lumbar spinal cord sections for the astrocytic and microglial activation markers GFAP (Figs. 3A and B) and IBA-1 (Figs. 3C and D), respectively. Glial fibrillary acidic protein levels were significantly higher only at day 54 after CAIA induction in both males and females as compared to controls. By contrast, we observed significantly higher levels of IBA-1 at both 15 and 54 days after CAIA when compared with saline controls (*P* < 0.001). IBA-1 can also stain other cell types within the myeloid lineage, particularly macrophages. In the context of our study, however, it is still an appropriate choice: in naive conditions, fate-mapping studies have convincingly demonstrated that microglia are the only resident myeloid cell in the central nervous system (see eg, Ref. 47 for review), and as discussed below, we do not observe peripheral immune cell infiltration after CAIA.

### 3.4. Intrathecal injection of minocycline or pentoxifylline reverses CAIA-induced mechanical hypersensitivity in male but not in female mice

**Figure 4.**
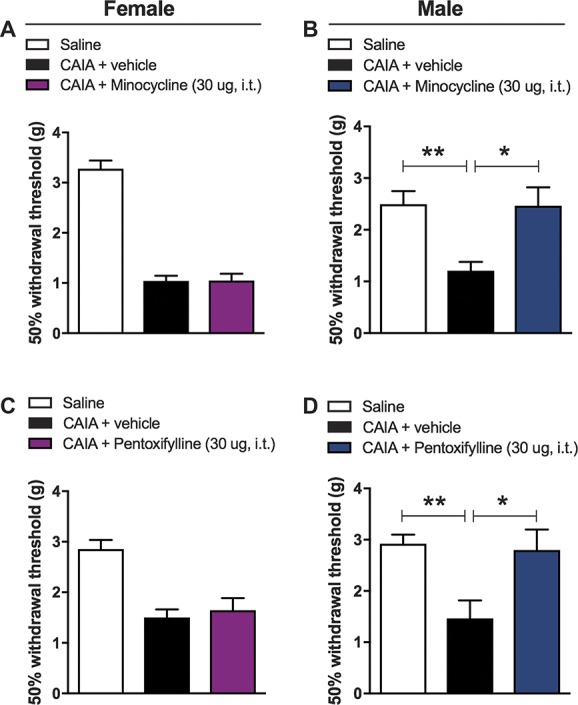
Intrathecal minocycline and pentoxifylline treatment reverses late phase mechanical hypersensitivity in the CAIA model in male mice only. Bar graphs showing the effects of spinal microglia inhibition on withdrawal thresholds of mice. Both minocycline and pentoxyfilline reversed mechanical hypersensitivity in males (B and D) but not in females (A and C) 6 hours after intrathecal administration. Data is presented as mean ± SEM, n = 6 per group; 1-way ANOVA, CAIA + vehicle vs CAIA + inhibitor, **P > 0.01, *P > 0.05. ANOVA, analysis of variance; CAIA, collagen antibody–induced arthritis.

Because both male and female glia displayed signs of reactivity in the CAIA model, we decided to investigate whether astrocytes and microglia could be causally involved in the maintenance of pain-like behaviour. For this purpose, we injected pentoxifylline or minocycline, drugs that are frequently used to inhibit spinal astrocytic or microglial actions, respectively, intrathecally in males and females during the postinflammatory phase (day 54-60) of the CAIA model. We found that, 6 hours after administration, both pentoxifylline and minocycline significantly reversed arthritis-induced withdrawal thresholds to control levels in males, but not in females (Fig. 4), suggesting that glia play sex-specific roles in arthritis-induced pain.

### 3.5. No differences in whole spinal cord microglial number or microglia-associated gene expression are observed between male and female mice

**Figure 5.**
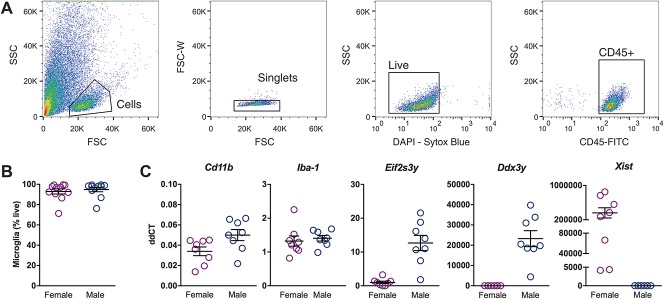
Microglia from whole naive spinal cord do not show prominent sex differences in number or expression level. (A) Gating strategy used to sort CD11b-positive live single immune cells. Shown are serial dot plots of the events recorded by the flow cytometer, with gates indicating chosen populations that are then displayed in the next adjacent plot. Sequentially, side scatter (SSC) vs forward scatter (FSC) to distinguish leukocytes from debris using their complexity and size; forward scatter width (FSC-W) vs area (FSC) to eliminate leukocyte doublets; FSC vs the live-dead cell marker Sytox blue to eliminate dead cells; and finally, FSC vs CD45, a marker for all immune cells. Only the events contained in this final CD45^+^ gate are taken forward for analysis of CD11b positivity. (B) No differences could be detected in the number of live microglia obtained from whole spinal cord of male and female mice. (C) Myeloid cell markers Iba-1 and CD11b did not show differential expression, whereas XY-linked genes *Xist*, *Eif2s3y* and *Ddx3y* were significantly more highly expressed in female or male mice, respectively.

Because FACS of astrocytes from adult spinal cord is still a challenging process, we focused on microglial cells for which there are several established sorting protocols.^9,17^ To examine whether there are sex-associated differences between microglia in naive mice, we compared the number of microglia isolated by FACS from the whole spinal cord of male and female mice (Fig. 5A) and the levels of *Cd11b* and *Iba-1* mRNA in these cells. We found no dimorphic differences in the relative number of CD45^+^, CD11b^+^ spinal microglial cells, their mean fluorescence intensity, or *Cd11b* and *Iba-1* mRNA expression when comparing naive mice of both sexes (Figs. 5B and C; Supplemental Figs. S1 and S2, available at http://links.lww.com/PAIN/A666). To attest that we could detect sex-associated differences, we measured the relative levels of the XY-linked genes *Ddx3y*, *Eif2s3*y, and *Xist*. We found significant differences between males and females, as expected (Fig. 5C). We also compared *Cd11b* and *Iba-1* mRNA levels in naive lumbar spinal cord homogenates from male and female mice of different strains. No difference in *Cd11b* and *Iba-1* gene expression between C57BL/6 and Balb/c males or C57BL/6 and Balb/c females was observed (Supplemental Fig. S4, available at http://links.lww.com/PAIN/A666).

### 3.6. No macrophage infiltration into the lumbar spinal cord is detected during the inflammatory phase of CAIA

**Figure 6.**
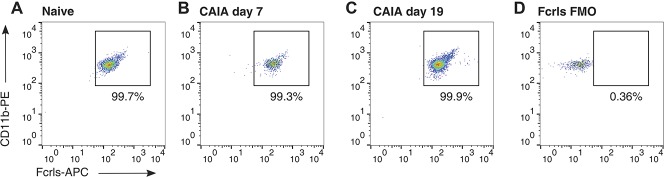
Macrophage infiltration into the lumbar dorsal horn is absent or negligible. Dot plots of myeloid cells (gated on live CD45^+^ positive singlets) from (A) naive and (B,C) CAIA animals isolated during the inflammatory phase (day 7 and day 19). Immune cells were stained for CD11b and Fcrls, a microglial cell marker not present in infiltrating macrophages. Both naive and CAIA groups have a similar proportion of CD11b^+^/Fcrls^+^ cells (over 99%) indicating that macrophage infiltration during the inflammatory phase of CAIA is negligible. (D) The rightmost panel displays a fluorescence-minus-one (FMO) control, where the Fcrls primary antibody was omitted from the staining panel. CAIA, collagen antibody–induced arthritis.

Next, we examined whether CAIA induction could lead to infiltration of macrophages because this would require separation of resident microglia and infiltrating macrophages in our samples. We focused on the lumbar dorsal horn, the region where spinal nociceptive signal processing related to the hind limbs takes place. We performed flow cytometry analyses on Percoll-isolated myeloid cells stained for CD45, CD11b, and Fcrls, a protein described to be expressed in resident microglia but not on macrophages.^35^ The percentage of CD45^+^, CD11b^+^, and Fcrls^+^ microglial cells amongst CD45^+^ immune cells was 99.2% and 99.9% at day 7 and day 19 of the CAIA inflammatory phase, respectively, similar to the 99.7% of its naive counterpart (Fig. 6). This indicates that macrophage infiltration in the lumbar dorsal horns is negligible, if any occurs at all. Based on this finding, we decided to sort microglial cells by selecting exclusively single live CD45 and CD11b positively stained cells.

### 3.7. In dorsal lumbar horns, microglial numbers are similar between saline and CAIA during the postinflammatory phase but are lower in females compared with males

**Figure 7.**
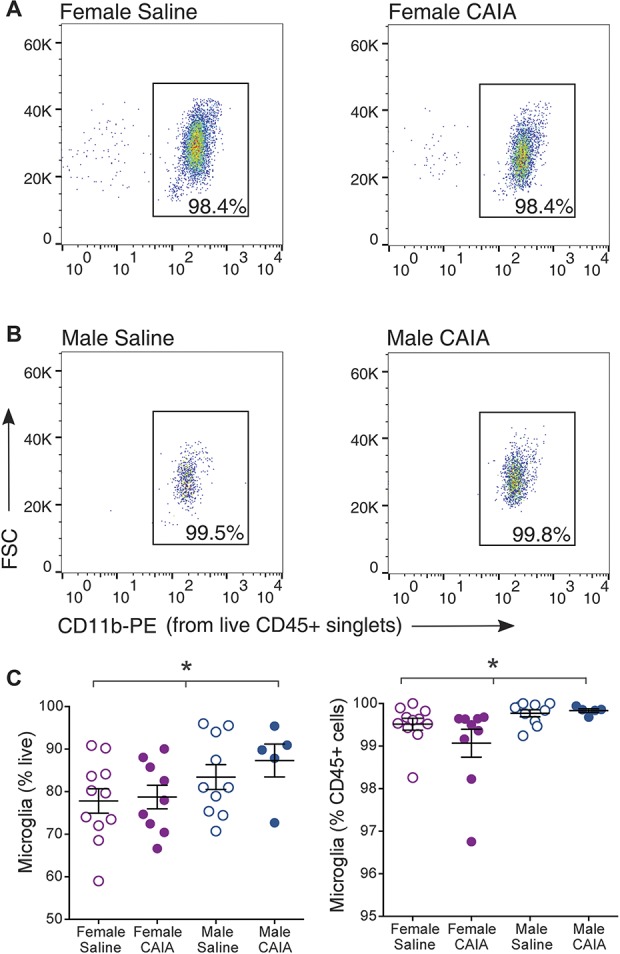
Flow cytometry analysis points to fewer dorsal horn microglial cells in females than males. Representative gating dot plots of CD45^+^ CD11b^+^ microglial cells from saline and CAIA (A) female and (B) male mice during the postinflammatory phase (day 54-day 60). (C) Percentage of CD45^+^ CD11b^+^ microglia amongst CD45^+^ immune cells. Females show a significant decrease in lumbar dorsal horn microglial and CD45^+^ cell numbers as compared to males (*P < 0.05, 1-way ANOVA), whereas the number of live cells does not differ (see Supplemental Fig. S1, available at http://links.lww.com/PAIN/A666). No differences were found between the saline and CAIA groups. Data are presented as mean ± SEM. ANOVA, analysis of variance; CAIA, collagen antibody–induced arthritis.

To gain a better insight into any possible differences between the saline and CAIA groups during the postinflammatory phase, we analyzed the flow cytometry data obtained from sorted CD45^+^, CD11b^+^ microglial cells from lumbar dorsal horns (Fig. 7). The percentage of microglia amongst CD45^+^ immune cells was mostly above 99% for all conditions (female saline: 99.5% ± 0.1; female CAIA: 99.1% ± 0.3; male saline: 99.8% ± 0.08; male CAIA: 99.8% ± 0.04), corroborating the observations made in naive and CAIA mice from the inflammatory phase (Fig. 6). The proportion of CD45^+^, CD11b^+^ microglia, CD45^+^ immune cells, and live cells did not significantly differ between saline and CAIA conditions (Fig. 7 and Supplemental Fig. S1, available at http://links.lww.com/PAIN/A666).

However, when the data were instead analyzed to study differences between males and females, we found a significantly lower percentage of female microglia amongst live cells (females: 78.2% ± 2.0; males: 84.7% ± 2.3; *P* < 0.05) and amongst CD45^+^ immune cells (females: 99.3% ± 0.2; males: 99.8% ± 0.06; *P* < 0.05) when compared with males (Fig. 7C). A lower proportion of CD45^+^ immune cells amongst live cells was also observed in females (females: 78.7% ± 12.0; males: 84.9% ± 2.3; *P* < 0.05), whereas the percentage of live cells amongst the total number of cells was similar in both sexes (Supplemental Fig. S1, available at http://links.lww.com/PAIN/A666).

### 3.8. RNA-sequencing reveals subtle transcriptional differences between male and female microglia but not between saline and postinflammatory CAIA

To investigate transcriptional differences between male and female microglia in the context of arthritis-induced pain, we performed RNA-seq on sorted CD45^+^, CD11b^+^ microglia from lumbar dorsal horns of saline, and CAIA mice during the postinflammatory phase. Quality control and alignment scores indicated that the data were of good quality (Supplemental Table 1, https://www.ncbi.nlm.nih.gov/geo/query/acc.cgi?acc=GSE108896; Supplemental Fig. S3, available at http://links.lww.com/PAIN/A666). We mapped an average of 37-M unique reads back onto the genome (min 27 M, max 46.5 M). Cufflinks and DESeq2 algorithms were used to generate normalized gene expression values Supplemental Table 2 (https://www.ncbi.nlm.nih.gov/geo/query/acc.cgi?acc=GSE108896) and to decipher differences in gene expression between conditions Supplemental Table 3 (https://www.ncbi.nlm.nih.gov/geo/query/acc.cgi?acc=GSE108896), respectively. We detected a total of 11,567 genes in lumbar dorsal horn microglia samples (FPKM ≥ 1 in most replicates in at least one condition) and found good overlap of microglial gene expression with previously published data sets: 85% of the genes we identified as present or absent were equally present or absent in at least 4 other RNA-seq studies performed on purified microglia Supplemental Table 4 (https://www.ncbi.nlm.nih.gov/geo/query/acc.cgi?acc=GSE108896).

**Figure 8.**
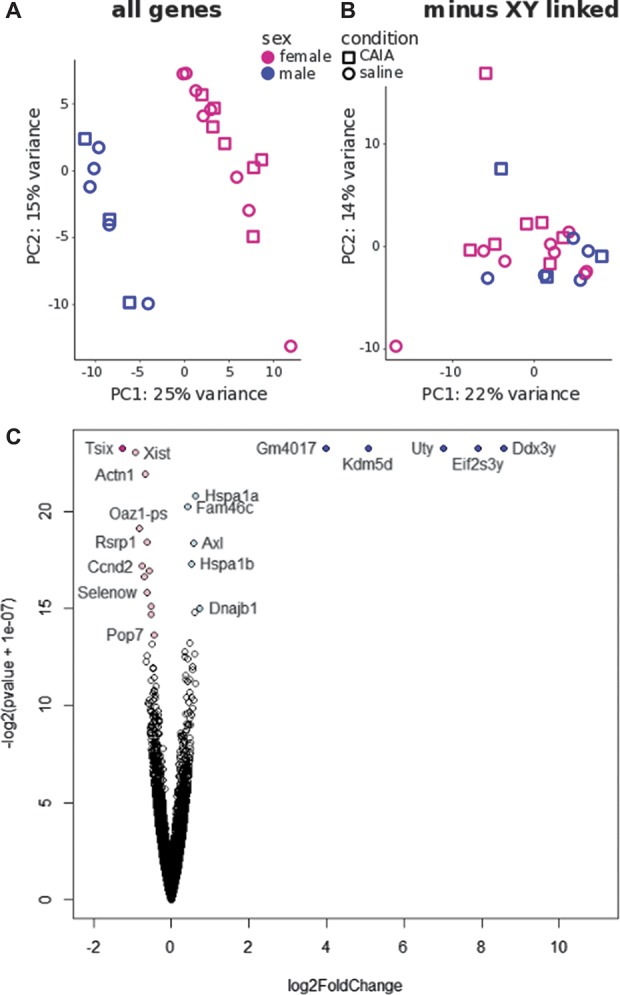
Principal component analysis reveals marked stratification between male and female microglia that are mostly due to XY-linked genetic differences. (A) PCA of male and female microglia samples from saline and postinflammatory CAIA groups. (B) PCA of male and female microglia samples in which significantly differentially expressed X- and Y-linked genes have been excluded from the analysis. (C) Volcano plot depicting differentially expressed genes between male and female microglia obtained using the Deseq2 algorithm. Colored dots represent significantly dysregulated genes at adjusted *P* < 0.05, logFC > 1 (dark blue for males, dark pink for females) and at adjusted *P* < 0.05, logFC < 1 (light blue for males, light pink for females). CAIA, collagen antibody–induced arthritis; PCA, principal component analysis.

To analyze any possible sex-dependent global variation on the sequenced microglia samples, we performed a principal component analysis. Interestingly, a pronounced separation was observed between the male and female microglial samples in the direction of the first principal component (25% variance), allowing the categorization of the samples in 2 well-defined groups. The overall variance across the first and second principal components was not markedly different when comparing saline and CAIA samples (Fig. 8A).

Using the DESeq2 algorithm, we found a total of 21 genes that were differentially expressed between male and female microglial cells at an adjusted *P* value (adj. *P*) <0.05 (Fig. 8C). Five of those (*Ddx3y*, *Eif2s3y*, *Uty*, *Xist*, and *Tsix*) were X- or Y-linked genes, which have also been described as differentially expressed in human and mouse brain.^4,58^ Their presence seems to account for most of the variance observed in the principal component analysis, as re-running the analysis without *Ddx3y*, *Eif2s3y*, *Uty*, *Xist*, and *Tsix* resulted in plots that were no longer separated into well-defined groups (Fig. 8B).

In contrast to differences between male and female microglia, differential expression analysis between CAIA vs saline samples did not reveal any convincing differences in gene expression between groups. Post hoc power calculations indicate that we are unlikely to have missed any large differences: our design gave us an 88% chance to detect a 2-fold change in expression in any of our 11,567 microglial genes and a more than 98% chance in the top 5700 genes (Supplemental Table 5, https://www.ncbi.nlm.nih.gov/geo/query/acc.cgi?acc=GSE108896). Only 2 genes (the pseudogene *Gm11761* and *Mesdc1*, a gene related to mesodermal patterning) passed multiple comparison corrections at adj. *P* < 0.1. Both of them showed extremely low expression, increasing the likelihood of these observations being due to statistical noise.

## 4. Discussion

There is an ongoing debate on the involvement of male and female microglia and astrocytes in chronic pain conditions. Although several groups have reported microglial contribution to pain states exclusively in males, other studies suggest that microglia might also play a role in females.^11,35,48^ It has been proposed that male mice use spinal microglia to mediate pain while females preferentially use the adaptive immune system, based on observations made in models of acute inflammatory and neuropathic pain.^48^ However, the detailed mechanisms of this process remain controversial.^29^

Equally in the case of astrocytes, both sex-dependent and independent contributions to chronic pain have been reported. Although pain reversal in spinal nerve injury and complete Freund's adjuvant models after fluorocitrate and propentofylline treatment occurs only in male mice,^48^ interfering with astrocytic activity using inhibitors for MAPKs, Cx43 hemichannel, and CXCR2 reduces mechanical hypersensitivity equally in male and female mice after chronic constriction injury but not in the formalin injection model of inflammatory pain.^11^

In the context of arthritis-induced pain, direct comparisons between male and female mice have so far been outstanding. It has been shown that minocycline can reduce joint nociception when injected intrathecally in male mice after antigen-induced arthritis induction,^42^ and that intrathecal administration of drugs targeting microglia (P2X7 and cathepsin S inhibitors) diminishes the development of mechanical hypersensitivity and attenuates microgliosis in female rats in the collagen-induced arthritis model.^35^ Furthermore, we have previously reported that blocking the action of spinal HMGB1, a factor that mediates nociceptive effects through TLR4 and induces glia activation, is able to reverse mechanical hypersensitivity in the inflammatory and late phases of the CAIA model both in male and female mice.^1^

In this study, we wanted to conduct a more thorough investigation of long-lasting arthritis-induced pain in both male and female mice. First off, we found some intrinsic differences related to the CAIA model. Although female mice were more likely to develop joint inflammation, mechanical hypersensitivity and spinal glial activation was similar across both sexes. In agreement with our previous published data,^49^ we found no upregulation of CGRP, SP, or galanin in the lumbar dorsal horns of male mice subjected to CAIA. Similarly, we found no changes in CGRP or SP spinal expression levels in female mice after CAIA, whereas a small but significant difference in galanin was observed between male and female mice subjected to CAIA during the postinflammatory phase. As the levels of galanin were slightly higher in male mice, the neuropeptide could in theory have contributed to arthritis-induced postinflammatory pain in a sex-specific manner. However, there was no marked increase in spinal galanin immunoreactivity over time in male mice. The link is therefore weak and potentially false-positive.

Interestingly, intrathecal administration of pentoxifylline reversed CAIA-induced mechanical hypersensitivity to basal levels in male, but not in female mice during the late phase. These findings support the existing evidence for a sex-dependent role of astrocytes in spinal pain processing,^48^ whereas it contradicts the recent study by Chen et al.^11^, which reports a male and female contribution of astrocytes in neuropathic pain despite an absent contribution to formalin-induced pain. Similarly, we also found dimorphic differences on minocycline treatment. Notably, intrathecal administration of minocycline reversed mechanical thresholds to control levels only in male mice during the late phase of the model. Although this finding is in line with studies using other models of chronic pain, it seems to contradict reports describing a possible role of female microglia in the maintenance of arthritis-induced pain. An intriguing thought could be that the mechanism of action of minocycline may differ between males and females. Thus, although both male and female microglia might be involved in pain maintenance, minocycline might only target male microglia in specific contexts. In fact, it is unclear how exactly this microglial inhibitor works, but it seems to affect some signalling pathways rather than completely inhibiting myeloid activation.^19^ Finally, it is worth mentioning that, although minocycline is frequently referred to as a selective microglial inhibitor after intrathecal delivery, it has effects on peripheral immune cells, neurons and other glial cells.^34^ Hence, it only seems prudent to interpret our minocycline data with caution, with regard to which cell type is ultimately mediating the effect we observed.

In light of this, we took a global approach to ascertain sex differences between resident male and female spinal microglia. Initially, we evaluated gross differences that could be present in the whole spinal cord of naive mice of both sexes. In agreement with recent work,^29^ we found little evidence of sex differences when examining relative numbers of sorted microglia or mRNA levels. As a positive control, we tested 3 XY-linked genes (*Ddx3y*, *Eif2s3y*, and *Xist*), which did show sexually dimorphic expression, as expected. In contrast to whole naive spinal cord, counts of microglia from lumbar dorsal horns showed a modest but statistically significant difference in numbers between male and female mice. Because experiments were performed in Balb/c vs C57BL6/J mice, this could be strain-specific effects. However, the basal levels of *Cd11b* and *Iba1* mRNA did not differ between the 2 strains. Thus, this sex difference might be region-specific: male and female microglial numbers have been reported to differ in subregions of the brain (hippocampus, amygdala, and parietal cortex),^46^ and the proliferation and activation status of microglia is increasingly believed to be under tight local control.^44^

Our earlier studies found that the late phase of the CAIA model resulted in spinal changes that were reminiscent of those reported in models of neuropathic pain.^49^ We had therefore predicted that microglia isolated from mice subjected to CAIA would show alterations in their transcriptional profile, which might resemble those observed after nerve injury.^17,51^ To our surprise, no convincing significant transcriptional differences were observed between CAIA and saline mice. Importantly, we were well-powered to detect changes that were at least 2-fold in size, whereas 1.5-fold changes would be much harder to reliably identify for anything, but the top 3000 most expressed genes. This may signify that microglial changes in CAIA are either subtle and/or localized to a very specific region, such as lamina I and II, making it difficult to detect any differences with the strategy used here. Alternatively, the microglial separation protocol used in this study might have impacted the microglial transcriptome, obscuring the detection of transcriptomic changes between the saline and CAIA conditions. Nevertheless, we believe this to be unlikely because studies using similar isolation techniques still detect differences between conditions.^12,16,37,51^ Moreover, a study that focused on the impact of isolation techniques on the transcriptome of microglia suggests that the transcriptome obtained from isolated quiescent or activated microglia closely reflects that of homeostatic microglia.^36^ Finally, our results could also indicate that prominent transcriptomic changes in microglia were transient and short-lasting because it has been recently suggested.^17,51^ Hence, microglial activation in CAIA and the possible contribution of microglia to arthritis-induced pain could be driven by other factors, such as changes in protein expression or epigenetic modifications. Another potential explanation, given our present findings, could be that the use of minocycline as a microglial inhibitor is misleading, and that microglia do not contribute to postinflammatory pain-like behaviour in this model at all. Instead, astrocytes may contribute to spinal sensitization in the CAIA model in male mice, whereas direct changes in neuronal factors such as galanin are the main drivers in females.

Taken together, these findings suggest that there are only subtle sex-dependent differences in microglial expression profiles, and if any, they are independent of treatment (saline and CAIA). To what extent these differences could explain the sexually dimorphic reversal of CAIA-induced pain after minocycline administration remains to be determined. Finally, we did not detect any transcriptional correlates to the immunohistochemical signs of increased microglial reactivity observed in the late phase of the CAIA model. It is likely that transcriptional changes are either subtle and highly localised and therefore difficult to identify with bulk isolation techniques or that other factors, such as changes in protein expression or epigenetic modifications, are at play.

## Conflict of interest statement

The authors have no conflict of interest to declare.

## Supplementary Material

SUPPLEMENTARY MATERIAL

## Appendix A. Supplemental digital content

Supplemental digital content associated with this article can be found online at http://links.lww.com/PAIN/A666. Supplemental tables can be downloaded at: https://www.ncbi.nlm.nih.gov/geo/query/acc.cgi?acc=GSE108896.
