# Supplementary material for: Exploring the transcriptome of resident spinal microglia after collagen antibody–induced arthritis
Source: Pain. 2018 Dec 28;160(1):224–36. doi: 10.1097/j.pain.0000000000001394 (PMC6319583; doi:10.1097/j.pain.0000000000001394)
Supplement: SUPPLEMENTARY MATERIAL [file jop-160-224-s001.docx]

**Abstract**

Recent studies have suggested a sexually dimorphic role of spinal glial cells in the maintenance of mechanical hypersensitivity in rodent models of chronic pain. We have used the collagen antibody-induced arthritis (CAIA) mouse model to examine differences between males and females in the context of spinal regulation of arthritis-induced pain. We have focused on the late phase of this model when joint inflammation has resolved but mechanical hypersensitivity persists. While the intensity of substance P, CGRP and galanin immunoreactivity in the spinal cord was not different from controls, the intensity of microglia (Iba-1) and astrocyte (GFAP) markers was elevated in both males and females. Intrathecal administration of the glial inhibitors minocycline and pentoxifylline reversed mechanical thresholds in male, but not female mice. We isolated resident microglia from the lumbar dorsal horns and observed a significantly lower number of microglial cells in females by flow cytometry analysis. However, while genome-wide RNA sequencing results pointed to several transcriptional differences between male and female microglia, no convincing differences were identified between control and CAIA groups.

Taken together, these findings suggest that there are subtle sex differences in microglial expression profiles independent of arthritis. Our experiments failed to identify the underlying mRNA correlates of microglial actions in the late phase of the CAIA model. It is likely that transcriptional changes are either subtle and highly localised and therefore difficult to identify with bulk isolation techniques or that other factors, such as changes in protein expression or epigenetic modifications are at play.

**Introduction**

Rheumatoid arthritis (RA) is an autoimmune disease that causes joint swelling and synovitis leading to progressive cartilage and bone destruction [[31](#_ENREF_31)]. Chronic pain is one of the most debilitating symptoms of this process [[22](#_ENREF_22),[52](#_ENREF_52)] and has long been attributed to peripheral inflammation. However, subgroups of patients report pain in spite of good medical control [[2](#_ENREF_2),[3](#_ENREF_3)]. Moreover, RA patients can develop a generalized increase in pain sensitivity at distant sites from the inflamed joint [[20](#_ENREF_20),[27](#_ENREF_27),[41](#_ENREF_41)], suggesting an involvement of the CNS [[32](#_ENREF_32)]. This notion is supported by studies reporting elevated cytokine levels in the CSF of patients and in animal models of RA [[25](#_ENREF_25),[26](#_ENREF_26),[35](#_ENREF_35)].

Microglia and astrocytes have emerged as crucial players in the maintenance of mechanical hypersensitivity [[1](#_ENREF_1),[6](#_ENREF_6),[14](#_ENREF_14),[17](#_ENREF_17),[35](#_ENREF_35),[56](#_ENREF_56)] which upon activation synthesize and release factors that facilitate neuronal excitability and transmission of nociception [[45](#_ENREF_45),[55](#_ENREF_55)]. Moreover, intrathecal delivery of glial inhibitors, such as minocycline or pentoxifylline, can prevent or reverse mechanical hypersensitivity in pain models [[15](#_ENREF_15),[23](#_ENREF_23)].

While arthritis and autoimmunity research has been fairly balanced in the consideration of both sexes, the majority of preclinical pain research has been performed in male animals. This is problematic in the context of studying pain in arthritis, since RA, like other autoimmune diseases, is more likely to occur in women [[24](#_ENREF_24)]. Furthermore, in an experimental setting, many authors [[5](#_ENREF_5),[33](#_ENREF_33)] – with some exceptions [[43](#_ENREF_43),[44](#_ENREF_44)] – have come to the conclusion that there are sexual dimorphisms in how nociception and potentially other sensory stimuli [[21](#_ENREF_21)] are processed. Recently, an effort has been made to include female rodents when studying models of chronic pain. As it is usual when scientific explorations are at an early stage, the results as well as their accompanying potential explanations have been mixed. A few laboratories have suggested that mechanical allodynia is reversed only in male but not female mice after intrathecal delivery of microglial inhibitors [[48](#_ENREF_48),[50](#_ENREF_50)]. The mechanisms underlying this phenomenon are still under debate [[29](#_ENREF_29),[48](#_ENREF_48)]. No clear sex differences have so far been reported in the context of pain in arthritis models [[1](#_ENREF_1),[35](#_ENREF_35)].

Here, we used the collagen antibody-induced arthritis (CAIA) model to investigate sexual dimorphisms in spinal signal transmission. In male mice, we have previously shown that spinal neuropeptide expression remains unchanged upon CAIA induction. Instead, we obtained evidence for the contribution of microglia and astrocytes in arthritis-induced pain in male mice. For instance, we demonstrated changes in microglial activation during the late phase of the CAIA model, when mechanical hypersensitivity persists but joint inflammation has resolved [[1](#_ENREF_1),[6](#_ENREF_6)]. Moreover, we showed that intrathecal injection of drugs that interfere with astrocytic activity (pentoxifylline or JNK inhibitors) were able to reverse CAIA-induced mechanical hypersensitivity in male mice [[6](#_ENREF_6)]. In the current study, we set out to confirm these findings – this time in both male and female mice. In addition to behavioural and immunohistochemical analyses, we also carried out genome-wide RNA-sequencing (RNA-seq) during the late phase of the CAIA model in order to further characterize the role of these cells in the context of arthritis-induced pain, and to identify targets that can be used for intervention. We hypothesized that microglia would play a similar role in females compared to males, given that it has been reported that this cell type is associated with pain-like behaviour in female rats subjected to collagen-induced arthritis [[14](#_ENREF_14)]. Interestingly, our results point towards a more complicated picture.

**Materials and Methods**

***Animals:*** Experimental procedures were carried out under ethical approval by the Northern Stockholm Animal Research Committee. Adult male and female Balb/cAnNRj (Janvier Labs), CBA (Harlan) (12-18 weeks old) and C57BL/6JRj (Janvier Labs) mice were used and housed in groups (3-5 mice per cage) in a temperature and humidity controlled environment with a 12h light/dark cycle and access to food and water *ad libitum*. Whole spinal cord experiments were carried out at King’s College London on 10 week old naïve male and female C57BL/6J mice in accordance with United Kingdom Home Office Legislation (Scientific Procedures Act 1986).

***Collagen antibody-induced arthritis (CAIA*):** For induction of arthritis, mice were injected intravenously (1.25-1.5 mg) with an anti-collagen type-II cocktail containing 5 different monoclonal autoantibodies (Chondrex) at day 0 followed by an intraperitoneal injection of LPS (25 μg in 100 μl; serotype 0111:B4; Chondrex, or 35 μg in 100 μl; serotype 055:B5; Sigma) at day 5.

***Scoring of arthritis:*** Joint inflammation of the fore and hind paws was evaluated visually as previously described [[6](#_ENREF_6)]. Briefly, 1 point was given for every inflamed toe or knuckle. Each paw, ankle or wrist was awarded 2.5 or 5 points if moderately or severely inflamed, respectively, resulting in a maximum arthritis score of 15 points per leg and 60 points per mouse. Animals that did not reach a minimum of 12 points in the hind paws at the peak of inflammation were excluded from the study.

***Mechanical hypersensitivity:*** Before testing, mice were acclimatized for 30-40 min in individual Plexiglas compartments with a wire mesh bottom. Mechanical hypersensitivity was assessed by applying calibrated Von Frey filaments (Optihair) of incremental force (0,03-3,30 g) to the plantar surface of the hind paw using the up-down method [[10](#_ENREF_10)]. The 50% paw withdrawal threshold (force at which the animal reacts 50% of the time) was calculated in grams for both hind paws and averaged. Three baseline testing sessions were performed on separate days and the average calculated for each animal. Results are presented as the average 50% withdrawal threshold for each group.

***Intrathecal injections:*** Female and male mice were injected intrathecally with minocycline (30 µg in 5 µl; M9511, Sigma), pentoxifylline (30 µg in 5 µl; P1784, Sigma) or saline (5 µl, vehicle control) under light isoflurane anaesthesia in the late phase (> 60 days after CAIA induction).

***Immunohistochemistry:*** At 15 and 54 days after anti-collagen type-II antibody cocktail or saline injection, male and female mice were deeply anaesthetized and transcardially perfused with saline (0.9% NaCl) followed by a fixative solution (4% paraformaldehyde with 0.2% picric acid in 0.16 M phosphate buffer). Lumbar spinal cords were dissected, post-fixed for 90 min at 4°C and cryoprotected with 10% sucrose in 0.1M phosphate buffer for 48 hours at 4°C. Spinal cords were then embedded in OCT compound (Tissue-Tek) and cut at 20 μm with a cryostat (Microm). Tissue sections were incubated overnight with primary antibodies against calcitonin gene-related peptide (CGRP) (1:32000, [[38](#_ENREF_38)]), substance P (SP) (1:4000, [[13](#_ENREF_13)]), galanin (1:4000, [[53](#_ENREF_53)]), glial fibrillary acidic protein (GFAP) (1:8000, Dako) and ionized calcium-binding adapter molecule 1 (IBA-1) (1:2000, Wako) at 4°C. Immunoreactivity was visualized with the TSA Plus kit (Perkin Elmer) as previously described [[8](#_ENREF_8)]. Images were captured with a LSM710 confocal laser-scanning microscope (Carl Zeiss) and the integrated signal intensity was measured after background subtraction in 3 sections per animal using Image J (NIH). Results are shown as the percentage change in signal intensity in CAIA compared to the saline group.

***Microglial isolation:*** Male and female mice (naïve, saline injected or subjected to CAIA) were sacrificed by anesthetic overdose and transcardially perfused with ice-cold Hank’s Balanced Salt Solution (HBSS). Spinal cords were flushed out using a HBSS filled-syringe inserted in the tail end of the spinal column. Lumbar dorsal horns were dissected and dounce homogenized in FACS buffer (0.4% BSA; 15mM HEPES; 2mM EDTA in HBSS) into a cell suspension that was centrifuged (430 x *g* for 30 min at 17°C) over a 37%/70% Percoll gradient (Sigma). Microglial cells were isolated from the interface and the total cell number determined using a haemocytometer. For each experiment, the lumbar dorsal horns of two mice were pooled into one Percoll gradient, constituting one *n* per sample group (2 x saline/naïve and 2 x CAIA).

***FACS & qRT-PCR of whole naïve spinal cord:*** Naïve male and female C57BL6/J mice (Harlan, 8 weeks old) were PBS perfused, and microglia were extracted in batches of 4 (n = 2, male and female) and stained for flow cytometry as described above. FACS was performed using a BD FACS Aria II Cell Sorter at the NIHR BRC flow core facility at King’s College London. Live, single CD45/CD11b double positive cells were collected into RLT buffer for later batch-controlled RNA extraction with a QIAGEN RNeasy® Micro Kit (74004) as described above. The RNA obtained was of high quality ranging from 75 pg/µl to 1490 pg/µl (average 451 pg/µl) with RIN values of 8.2-10 (average 9). The material was amplified using the Smart-seq2 protocol [[39](#_ENREF_39),[40](#_ENREF_40)], according to the instructions provided by the authors. qRT-PCR was conducted using primer pairs listed in Table 1, after having confirmed their efficiency and specificity (via melt curve and agarose gel analysis). In a separate experiment, additional qRT-PCR was performed on the lumbar part of spinal cords from naïve male and female (n=11-13/group) C57BL6/J and Balb/cAnNRj mice (Janvier, 12-18 weeks old). Flash frozen tissue was homogenized with bead Tissue Lyser (Qiagen) in Trizol (Thermo Fischer Scientific) and mRNA was extracted according to the manufacturer’s protocol. The cDNA was prepared by reverse transcription and subjected to qPCR with ABI 7900HT system, using TaqMan probes (Applied Biosystems): *Iba-1* (*Mm00479862_g1), Cd11b (Mm00434455_m1), GAPDH (Mm99999915_g1).* Threshold cycle values in each sample were used to calculate the number of cell equivalents in the test samples using the standard curve method [[7](#_ENREF_7)]. The data was normalized to GAPDH mRNA levels and expressed as relative expression units (REU).

***FACS of lumbar dorsal horns from CAIA and control mice:*** Microglial cells were centrifuged (230 x *g* for 3 min at 4°C) and stained with a monoclonal rat anti-Fcrls antibody (1:300; clone 4G11, kindly donated by Dr. Oleg Butovsky, Harvard, USA) for 20 min on ice. After a wash with FACS buffer and a second spin, cells were incubated for 20 min on ice with a goat anti-rat IgG secondary antibody conjugated to APC (1:400; clone Poly4054; Biolegend). Cells were washed and spun again, followed by staining with directly conjugated rat anti-mouse CD45-PE (1:400; clone 30-F11; Biolegend) and rat anti-mouse CD11b-FITC (1:400; clone 3A33; Abcam) antibodies for 20 min on ice. After washing, spinning and staining dead cells with Sytox™ blue (1:1500; Life Technologies), cells were sorted with a BD Influx™ flow cytometer (core facility at the Center for Molecular Medicine, Karolinska Institutet) and purified microglia were collected and lysed in RLT buffer (Qiagen, 79216) containing 1% β-mercaptoethanol. Unstained cells and single staining controls (Sytox™ blue stained dead cells and BD Comp beads [BD Biosciences] for FITC, PE and APC fluorochromes) were used for compensation. To aid the gating, we used a fluorescence-minus-one control, where the Fcrls primary antibody was omitted to control for unspecific fluorescence of the APC secondary antibody. Naïve and CAIA injected animals in the inflammatory phase (day 9 or day 17) were stained for all three markers (Fcrls, CD45, CD11b) whereas saline and CAIA injected animals in the post-inflammatory phase (days 55-65) were only stained for CD45 and CD11b. For data analysis in FlowJo, gates were kept constant across conditions in the same experimental set (saline/naïve vs. CAIA). On average, 3200 microglia were sorted for these experiments in group-matched batches. Only 3 out of 31 samples contained less than 1000 cells.

***RNA extraction:*** RNA from lumbar dorsal horn sorted microglial cells was extracted using a QIAGEN RNeasy® Micro Kit (74004) according to the manufacturer’s instructions with minor modifications. If sorted samples contained less than 2,000 cells, then two samples from the same condition were pooled into one, constituting one *n* per sample group (2-4 x saline and 2-4 x CAIA mice). RNA quantity and integrity were assessed with a Bioanalyzer using Pico Chips (Agilent Technologies).

***Sequencing:*** cDNA library preparation and RNA sequencing were performed by the High Throughput Genomics Group at the Wellcome Trust Centre for Human Genetics (Oxford University). The cDNA libraries were prepared with a SMARTer® Ultra® Low Input RNA Kit (Clontech). Samples were amplified and multiplexed on an Illumina HiSeq4000 platform to yield 75bp fragments at a depth of at least 27 million reads per sample. Each sample sequenced (n=23) contained lumbar dorsal horn sorted microglia obtained from a pool of 2-4 saline or CAIA mice.

***RNA-Seq Data Analysis:*** Relevant quality control was performed using the RSeQC algorithm [[57](#_ENREF_57)]. In particular, we checked for gene body coverage, since SMARTer library prep can result in significant 3’ bias. Reads were aligned to the mm10 mouse genome using STAR [[18](#_ENREF_18)] (default parameters plus: --outFilterMultimapNmax 1 --clip3pAdapterSeq "TGGTATCAACGCAGAGTAC"). Alignment rates were between 83% and 92% (89% on average). FPKM values were obtained using the cufflinks algorithm [[54](#_ENREF_54)] on the Galaxy Freiburg server. Genes with an FPKM value of at least 1 in a majority of samples per group were considered expressed. For differential gene expression we generated count data with the aid of the feature counts algorithm [[28](#_ENREF_28)] and fed them into the Deseq2 algorithm [[30](#_ENREF_30)] in R. Raw and processed data are available under GEO accession GSE108896.

***Statistics:*** For data besides RNA-seq, differences between two groups were analysed with a two-tailed unpaired student’s t-test. Comparisons between more than two groups were performed with one-way analysis of variance (ANOVA) for one independent variable or two-way ANOVA for two independent variables. Multiple comparisons were adjusted with Bonferroni post-hoc test. Data is shown as mean ± standard error of the mean (SEM).

**Results**

**CAIA induces transient joint inflammation and persistent mechanical hypersensitivity in male and female mice**

Adult male and female mice were subjected to CAIA by an intravenous (i.v.) injection of a collagen type-II antibody cocktail on day 0, followed by an intraperitoneal (i.p.) injection of LPS on day 5 to boost and synchronize the arthritogenic response. We found significant differences in CAIA incidence between males and females; while 90% (27/30) of females developed CAIA, only 53.5% (23/43) of males did. Male and female mice that developed CAIA presented visually apparent joint swelling and redness from day 6, reaching a peak in arthritis score around day 12 and gradually returning to baseline levels around day 30 (Fig. 1A). While joint inflammation was transient in all mice, mechanical hypersensitivity, which was present from day 6, persisted up until the last testing point at day 52 (p<0.05 to p<0.001 on days 6-52 compared to saline controls) (Fig. 1B). We refer to the period characterized by joint swelling and mechanical hypersensitivity as the “inflammatory phase”, and the period where joint inflammation has resolved but mechanical hypersensitivity persists as the “post-inflammatory phase” or “late phase”. Notably, saline matched controls did not present any signs of inflammation or mechanical hypersensitivity at any time point.

**CAIA does not alter expression levels of spinal CGRP, SP or galanin**

Previously, we have shown that male mice subjected to CAIA do not display spinal changes in neuropeptides associated with pain (CGRP, SP and galanin) during the inflammatory and post-inflammatory phases of this model. To investigate if female mice show altered expression of these neuropeptides after CAIA induction, we compared male and female lumbar spinal cords at the peak of the inflammatory phase (15 days) and during the post-inflammatory phase (54 days). In line with our previous work, we found no change in expression levels for CGRP, SP nor galanin in male mice at the time points examined after CAIA induction (Fig. 2). Nevertheless, comparing male and female mice in the late phase, we found that galanin expression levels were modestly but statistically higher in male compared to female CAIA mice in the late phase (Fig. 2C). However, there was no significant difference in spinal galanin levels between control and CAIA mice, independent on sex and time point. No changes in CGRP or SP expression were observed in female mice after CAIA induction nor in saline controls from both sexes.

**CAIA leads to an increase in spinal GFAP and IBA-1 immunoreactivity in male and female mice**

We have previously shown that male mice subjected to CAIA display signs of microglial activation both in the inflammatory and late phases whereas they only show increased levels of GFAP in the post-inflammatory phase of the model [[1](#_ENREF_1),[6](#_ENREF_6)]. To understand whether female mice show the same pattern of glial reactivity, we stained lumbar spinal cord sections for the astrocytic and microglial activation markers GFAP (Fig. 3A,B) and IBA-1 (Fig. 3C,D), respectively. GFAP levels were significantly higher only at day 54 after CAIA induction in both males and females as compared to controls. In contrast, we observed significantly higher levels of IBA-1 at both 15 and 54 days after CAIA when compared to saline controls (p<0.001). IBA-1 can also stain other cell types within the myeloid lineage, particularly macrophages. In the context of our study, however, it is still an appropriate choice: in naïve conditions, fate-mapping studies have convincingly demonstrated that microglia are the only resident myeloid cell in the CNS (see e.g. [[47](#_ENREF_47)] for review) and, as discussed below, we do not observe peripheral immune cell infiltration after CAIA.

**Intrathecal injection of minocycline or pentoxifylline reverses CAIA-induced mechanical hypersensitivity in male but not female mice**

Since both male and female glia displayed signs of reactivity in the CAIA model, we decided to investigate whether astrocytes and microglia could be causally involved in the maintenance of pain-like behavior. For this purpose, we injected pentoxifylline or minocycline, drugs that are frequently used to inhibit spinal astrocytic or microglial actions, respectively, intrathecally in males and females during the post-inflammatory phase (day 54-60) of the CAIA model. We found that, 6 hours after administration, both pentoxifylline and minocycline significantly reversed arthritis-induced withdrawal thresholds to control levels in males, but not females (Fig. 4), suggesting that glia play sex-specific roles in arthritis-induced pain.

**No differences in whole spinal cord microglial number or microglia-associated gene expression are observed between male and female mice**

Since fluorescence-activated cell sorting (FACS) of astrocytes from adult spinal cord is still a challenging process, we focused on microglial cells for which there are several established sorting protocols [[9](#_ENREF_9),[17](#_ENREF_17)]. To examine if there are sex-associated differences between microglia in naive mice, we compared the number of microglia isolated by FACS from the whole spinal cord of male and female mice (Fig. 5A) and the levels of *Cd11b* and *Iba-1* mRNA in these cells. We found no dimorphic differences in the relative number of CD45^+^, CD11b^+^ spinal microglial cells, their mean fluorescence intensity or Cd11b or Iba-1 mRNA expression when comparing naïve mice of both sexes (Fig. 5B, C; Suppl. Figs. S1 & S2, available at http://links.lww.com/PAIN/A666). In order to attest that we could detect sex-associated differences we measured the relative levels of the XY linked genes *Ddx3y*, *Eif2s3*y and *Xist*. We found significant differences between males and females, as expected (Fig. 5C). We also compared *Cd11b* and *Iba-1* mRNA levels in naïve lumbar spinal cord homogenates from male and female mice of different strains. No difference in *Cd11b* and *Iba-1* gene expression between C57BL/6 and Balb/c males or C57BL/6 and Balb/c females was observed (Suppl. Fig. S4, available at http://links.lww.com/PAIN/A666).

**No macrophage infiltration into the lumbar spinal cord is detected during the inflammatory phase of CAIA**

Next, we examined if CAIA induction could lead to infiltration of macrophages, as this would require separation of resident microglia and infiltrating macrophages in our samples. We focused on the lumbar dorsal horn, the region where spinal nociceptive signal processing related to the hind limbs takes place. We performed flow cytometry analyses on Percoll-isolated myeloid cells stained for CD45, CD11b and Fcrls, a protein described to be expressed in resident microglia but not on macrophages [35]. The percentage of CD45^+^, CD11b^+^ and Fcrls^+^ microglial cells amongst CD45^+^ immune cells was 99.2% and 99.9% at day 7 and day 19 of the CAIA inflammatory phase, respectively, similar to the 99.7% of its naïve counterpart (Fig. 6). This indicates that macrophage infiltration in the lumbar dorsal horns is negligible, if any occurs at all. Based on this finding, we decided to sort microglial cells by selecting exclusively single live CD45 and CD11b positively stained cells.

**In dorsal lumbar horns, microglial numbers are similar between saline and CAIA during the post-inflammatory phase but are lower in females compared to males**

To gain a better insight into any possible differences between the saline and CAIA groups during the post-inflammatory phase, we analyzed the flow cytometry data obtained from sorted CD45^+^, CD11b^+^ microglial cells from lumbar dorsal horns (Fig. 7). The percentage of microglia amongst CD45^+^ immune cells was mostly above 99% for all conditions (female saline: 99.5% ± 0.1; female CAIA: 99.1% ± 0.3; male saline: 99.8% ± 0.08; male CAIA: 99.8% ± 0.04), corroborating the observations made in naïve and CAIA mice from the inflammatory phase (Fig. 6). The proportion of CD45^+^, CD11b^+^ microglia, CD45^+^ immune cells and live cells did not significantly differ between saline and CAIA conditions (Fig. 7 and Suppl. Fig. S1, available at http://links.lww.com/PAIN/A666).

However, when the data were instead analyzed to study differences between males and females, we found a significantly lower percentage of female microglia amongst live cells (females: 78.2% ± 2.0; males: 84.7% ± 2.3; p<0.05) and amongst CD45^+^ immune cells (females: 99.3% ± 0.2; males: 99.8% ± 0.06; p<0.05) when compared to males (Fig. 7C). A lower proportion of CD45^+^ immune cells amongst live cells was also observed in females (females: 78.7% ± 12.0; males: 84.9% ± 2.3; p<0.05), while the percentage of live cells amongst the total number of cells was similar in both sexes (Suppl. Fig. S1, available at http://links.lww.com/PAIN/A666).

**RNA-sequencing reveals subtle transcriptional differences between male and female microglia but not between saline and post-inflammatory CAIA**

In order to investigate transcriptional differences between male and female microglia in the context of arthritis-induced pain, we performed RNA-seq on sorted CD45^+^, CD11b^+^ microglia from lumbar dorsal horns of saline and CAIA mice during the post-inflammatory phase. Quality control and alignment scores indicated that the data were of good quality (Suppl. Fig. S3, available at http://links.lww.com/PAIN/A666). We mapped an average of 37M unique reads back onto the genome (min 27M, max 46.5M). Cufflinks and DESeq2 algorithms were used to generate normalized gene expression values and to decipher differences in gene expression between conditions, respectively. We detected a total of 11,567 genes in lumbar dorsal horn microglia samples (FPKM >=1 in the majority of replicates in at least one condition) and found good overlap of microglial gene expression with previously published datasets: 85% of the genes we identified as present or absent were equally present or absent in at least 4 other RNA-seq studies performed on purified microglia.

To analyze any possible sex-dependent global variation on the sequenced microglia samples, we performed a principal component analysis (PCA). Interestingly, a pronounced separation was observed between the male and female microglial samples in the direction of the first principal component (25% variance), allowing the categorization of the samples in two well-defined groups. The overall variance across the first and second principal components was not markedly different when comparing saline and CAIA samples (Fig. 8A).

Using the DESeq2 algorithm, we found a total of 21 genes that were differentially expressed between male and female microglial cells at an adjusted P value (adj. p) <0.05 (Fig. 8C). Five of those (Ddx3y, Eif2s3y, Uty, Xist and Tsix) were X or Y linked genes, which have also been described as differentially expressed in human and mouse brain [[4](#_ENREF_4),[58](#_ENREF_58)]. Their presence appears to account for most of the variance observed in the PCA, as re-running the analysis without Ddx3y, Eif2s3y, Uty, Xist and Tsix resulted in plots that were no longer separated into well-defined groups (Fig. 8B).

In contrast to differences between male and female microglia, differential expression analysis between CAIA vs. saline samples did not reveal any convincing differences in gene expression between groups. Posthoc power calculations indicate that we are unlikely to have missed any large differences: our design gave us an 88% chance to detect a 2-fold change in expression in any of our 11,567 microglial genes and a more than 98% chance in the top 5,700 genes. Only two genes (the pseudo gene Gm11761 and Mesdc1, a gene related to mesodermal patterning) passed multiple comparison corrections at adj. p < 0.1. Both of them showed extremely low expression, increasing the likelihood of these observations being due to statistical noise.

**Discussion**

There is an ongoing debate on the involvement of male and female microglia and astrocytes in chronic pain conditions. While several groups have reported microglial contribution to pain states exclusively in males, other studies suggest that microglia might also play a role in females [[11](#_ENREF_11),[35](#_ENREF_35),[48](#_ENREF_48)]. It has been proposed that male mice utilize spinal microglia to mediate pain while females preferentially use the adaptive immune system, based on observations made in models of acute inflammatory and neuropathic pain [[48](#_ENREF_48)]. However, the detailed mechanisms of this process remain controversial [[29](#_ENREF_29)].

Equally in the case of astrocytes, both sex-dependent and independent contributions to chronic pain have been reported. While pain reversal in spinal nerve injury (SNI) and complete Freund’s adjuvant (CFA) models after fluorocitrate and propentofylline treatment occurs only in male mice [[48](#_ENREF_48)], interfering with astrocytic activity using inhibitors for MAPKs, Cx43 hemichannel and CXCR2 reduces mechanical hypersensitivity equally in male and female mice after chronic constriction injury (CCI) but not in the formalin-injection model of inflammatory pain [[11](#_ENREF_11)].

In the context of arthritis-induced pain, direct comparisons between male and female mice have so far been outstanding. It has been shown that minocycline can reduce joint nociception when injected intrathecally in male mice after antigen-induced arthritis induction (AIA) [[42](#_ENREF_42)], and that intrathecal administration of drugs targeting microglia (P2X7 and cathepsin S inhibitors) diminishes the development of mechanical hypersensitivity and attenuates microgliosis in female rats in the collagen-induced arthritis (CIA) model [[35](#_ENREF_35)]. Furthermore, we have previously reported that blocking the action of spinal HMGB1, a factor that mediates nociceptive effects via TLR4 and induces glia activation, is able to reverse mechanical hypersensitivity in the inflammatory and late phases of the CAIA model both in male and female mice [[1](#_ENREF_1)].

In the present study, we wanted to conduct a more thorough investigation of long-lasting arthritis-induced pain in both male and female mice. First off, we found some intrinsic differences related to the CAIA model. While female mice were more likely to develop joint inflammation, mechanical hypersensitivity and spinal glial activation was similar across both sexes. In agreement with our previous published data [[49](#_ENREF_49)], we found no upregulation of CGRP, SP or galanin in the lumbar dorsal horns of male mice subjected to CAIA. Similarly, we found no changes in CGRP or SP spinal expression levels in female mice after CAIA whereas a small but significant difference in galanin was observed between male and female mice subjected to CAIA during the post-inflammatory phase. As the levels of galanin were slightly higher in male mice, the neuropeptide could in theory have contributed to arthritis-induced post-inflammatory pain in a sex-specific manner. However, there was no marked increase in spinal galanin immunoreactivity over time in male mice. The link is therefore weak and potentially false-positive.

Interestingly, intrathecal administration of pentoxifylline reversed CAIA-induced mechanical hypersensitivity to basal levels in male, but not in female mice during the late phase. These findings support the existing evidence for a sex-dependent role of astrocytes in spinal pain processing [[48](#_ENREF_48)] while it contradicts the recent study by Chen et al; which reports a male and female contribution of astrocytes in neuropathic pain despite an absent contribution to formalin-induced pain [[11](#_ENREF_11)]. Similarly, we also found dimorphic differences upon minocycline treatment. Notably, intrathecal administration of minocycline reversed mechanical thresholds to control levels only in male mice during the late phase of the model. While this finding is in line with studies using other models of chronic pain, it seems to contradict reports describing a possible role of female microglia in the maintenance of arthritis-induced pain. An intriguing thought could be that the mechanism of action of minocycline may differ between males and females. Thus, while both male and female microglia might be involved in pain maintenance, minocycline might only target male microglia in specific contexts. In fact, it is unclear how exactly this microglial inhibitor works, but it seems to affect some signalling pathways rather than completely inhibiting myeloid activation [[19](#_ENREF_19)]. Finally, it is worth mentioning that, although minocycline is frequently referred to as a selective microglial inhibitor after intrathecal delivery, it has effects on peripheral immune cells, neurons and other glial cells [[34](#_ENREF_34)]. Hence, it only seems prudent to interpret our minocycline data with caution, with regards to which cell type is ultimately mediating the effect we observed.

In light of this, we took a global approach to ascertain sex-differences between resident male and female spinal microglia. Initially, we evaluated gross differences that could be present in the whole spinal cord of naïve mice of both sexes. In agreement with recent work [[29](#_ENREF_29)], we found little evidence of sex differences when examining relative numbers of sorted microglia or mRNA levels. As a positive control, we tested three XY linked genes (Ddx3y, Eif2s3y, Xist), which did show sexually dimorphic expression, as expected. In contrast to whole naïve spinal cord, counts of microglia from lumbar dorsal horns showed a modest, but statistically significant difference in numbers between male and female mice. Since experiments were carried out in Balb/c vs. C57BL6/J mice, this could be strain specific effects. However, the basal levels of *Cd11b* and *Iba1* mRNA did not differ between the two strains. Thus, this sex difference might be region specific: male and female microglial numbers have been reported to differ in subregions of the brain (hippocampus, amygdala and parietal cortex) [[46](#_ENREF_46)], and the proliferation and activation status of microglia is increasingly thought to be under tight local control [[44](#_ENREF_44)].

Our earlier studies found that the late phase of the CAIA model resulted in spinal changes that were reminiscent of those reported in models of neuropathic pain [[49](#_ENREF_49)]. We had therefore predicted that microglia isolated from mice subjected to CAIA would show alterations in their transcriptional profile, which might resemble those observed after nerve injury [[17](#_ENREF_17),[51](#_ENREF_51)]. To our surprise, no convincing significant transcriptional differences were observed between CAIA and saline mice. Importantly, we were well powered to detect changes that were at least 2-fold in size, while 1.5-fold changes would be much harder to reliably identify for anything but the top 3,000 most expressed genes. This may signify that microglial changes in CAIA are either subtle and/or localized to a very specific region, such as lamina I and II, making it difficult to detect any differences with the strategy employed here. Alternatively, the microglial separation protocol used in this study might have impacted the microglial transcriptome, obscuring the detection of transcriptomic changes between the saline and CAIA conditions. Nevertheless, we believe this to be unlikely, since studies using similar isolation techniques still detect differences between conditions [[12](#_ENREF_12),[16](#_ENREF_16),[37](#_ENREF_37),[51](#_ENREF_51)]. Moreover, a study which focused on the impact of isolation techniques on the transcriptome of microglia suggests that the transcriptome obtained from isolated quiescent or activated microglia closely reflects that of homeostatic microglia [[36](#_ENREF_36)]. Finally, our results could also indicate that prominent transcriptomic changes in microglia were transient and short-lasting, as it has been recently suggested [[17](#_ENREF_17),[51](#_ENREF_51)]. Hence, microglial activation in CAIA and the possible contribution of microglia to arthritis-induced pain could be driven by other factors, such as changes in protein expression or epigenetic modifications. Another potential explanation, given our present findings, could be that the use of minocycline as a microglial inhibitor is misleading and that microglia do not contribute to post-inflammatory pain-like behaviour in this model at all. Instead, astrocytes may contribute to spinal sensitization in the CAIA model in male mice while direct changes in neuronal factors such as galanin are the main drivers in females.

Taken together, these findings suggest that there are only subtle sex-dependent differences in microglial expression profiles, and if any, they are independent of treatment (saline, CAIA). To what extent these differences could explain the sexually dimorphic reversal of CAIA-induced pain after minocycline administration remains to be determined. Finally, we did not detect any transcriptional correlates to the immunohistochemical signs of increased microglial reactivity observed in the late phase of the CAIA model. It is likely that transcriptional changes are either subtle and highly localised and therefore difficult to identify with bulk isolation techniques or that other factors, such as changes in protein expression or epigenetic modifications are at play.

**Acknowledgements:**

The study was supported by the Swedish Research Council (CIS, TH), Knut and Alice Wallenberg Foundation (CIS), the Swedish Foundation for Strategic Research (CIS, ZWH), William K Bowes Foundation (CIS), the Karolinska Institute Foundations (TFZ), the MRC ERA-NET Neuron grant (MR/M501785/1), the European Union’s Seventh Framework Programme (FP7/2007 - 2013) under grant agreement No. 602919 (GLORIA) and Horizon 2020 research and innovation program under the Marie Skłodowska-Curie grant agreement No. 642720 (BonePain). We would like to thank Annika van Vollenhoven and the flow cytometry facility at CMM (Karolinska Institutet) for assistance in cell sorting experiments. FACS experiments at King’s College London were supported by the National Institute for Health Research (NIHR) Biomedical Research Centre based at Guy’s and St. Thomas’ NHS Foundation Trust and King’s College London. The views expressed are those of the author(s) and not necessarily those of the NHS, the NIHR, or the Department of Health.

We also thank the High-Throughput Genomics Group at the Wellcome Trust Centre for Human Genetics (funded by Wellcome Trust grant 090532/Z/09/Z) for the generation of the sequencing data, and Georgios Baskozos for kindly sharing some of his scripts.

Finally, the authors acknowledge the support of the Freiburg Galaxy Team: Person X and Prof. Rolf Backofen, Bioinformatics, University of Freiburg, Germany funded by Collaborative Research Centre 992 Medical Epigenetics (DFG grant SFB 992/1 2012) and German Federal Ministry of Education and Research (BMBF grants 031 A538A/A538C RBC, 031L0101B/031L0101C de.NBI-epi, 031L0106 de.STAIR (de.NBI).

The authors have no conflict of interest to declare.

**Author contributions:**

FD, TFZ, ZWH, XJX, TH and CIS designed the study and discussed the outcome of experiments. TFZ performed microglial isolation and sorting experiments, RNA extractions for sequencing and some data analysis. FD analysed the flow cytometry and sequencing data. JE provided technical assistance for flow cytometry under supervision of JL. NA, KS and AJ carried out behavioural experiments. TG and JS performed the immunohistochemical studies and analyses. ZH carried out naïve whole spinal cord sorting experiments for qPCR. AJ performed qPCR analyses in the naïve lumbar spinal cords. TFZ wrote the initial manuscript. FD, CIS, KS, AJ and JE provided critical feedback and revised the original manuscript.

**References**

[1] Agalave NM, Larsson M, Abdelmoaty S, Su J, Baharpoor A, Lundback P, Palmblad K, Andersson U, Harris H, Svensson CI. Spinal HMGB1 induces TLR4-mediated long-lasting hypersensitivity and glial activation and regulates pain-like behavior in experimental arthritis. Pain 2014;155(9):1802-1813.

[2] Altawil R, Saevarsdottir S, Wedren S, Alfredsson L, Klareskog L, Lampa J. Remaining Pain in Early Rheumatoid Arthritis Patients Treated With Methotrexate. Arthritis Care Res (Hoboken) 2016;68(8):1061-1068.

[3] Andersson MLE, Forslind K, Hafstrom I, Group BS. Patients with Early Rheumatoid Arthritis in the 2000s Have Equal Disability and Pain Despite Less Disease Activity Compared with the 1990s: Data from the BARFOT Study over 8 Years. J Rheumatol 2017;44(6):723-731.

[4] Armoskus C, Moreira D, Bollinger K, Jimenez O, Taniguchi S, Tsai HW. Identification of sexually dimorphic genes in the neonatal mouse cortex and hippocampus. Brain Res 2014;1562:23-38.

[5] Bartley EJ, Fillingim RB. Sex differences in pain: a brief review of clinical and experimental findings. British journal of anaesthesia 2013;111(1):52-58.

[6] Bas DB, Su J, Sandor K, Agalave NM, Lundberg J, Codeluppi S, Baharpoor A, Nandakumar KS, Holmdahl R, Svensson CI. Collagen antibody-induced arthritis evokes persistent pain with spinal glial involvement and transient prostaglandin dependency. Arthritis and rheumatism 2012;64(12):3886-3896.

[7] Boyle DL, Rosengren S, Bugbee W, Kavanaugh A, Firestein GS. Quantitative biomarker analysis of synovial gene expression by real-time PCR. Arthritis Res Ther 2003;5(6):R352-360.

[8] Brumovsky P, Stanic D, Shuster S, Herzog H, Villar M, Hokfelt T. Neuropeptide Y2 receptor protein is present in peptidergic and nonpeptidergic primary sensory neurons of the mouse. J Comp Neurol 2005;489(3):328-348.

[9] Butovsky O, Jedrychowski MP, Moore CS, Cialic R, Lanser AJ, Gabriely G, Koeglsperger T, Dake B, Wu PM, Doykan CE, Fanek Z, Liu L, Chen Z, Rothstein JD, Ransohoff RM, Gygi SP, Antel JP, Weiner HL. Identification of a unique TGF-beta-dependent molecular and functional signature in microglia. Nat Neurosci 2014;17(1):131-143.

[10] Chaplan SR, Bach FW, Pogrel JW, Chung JM, Yaksh TL. Quantitative assessment of tactile allodynia in the rat paw. J Neurosci Methods 1994;53(1):55-63.

[11] Chen G, Luo X, Qadri MY, Berta T, Ji RR. Sex-Dependent Glial Signaling in Pathological Pain: Distinct Roles of Spinal Microglia and Astrocytes. Neurosci Bull 2017.

[12] Chiu IM, Morimoto ET, Goodarzi H, Liao JT, O'Keeffe S, Phatnani HP, Muratet M, Carroll MC, Levy S, Tavazoie S, Myers RM, Maniatis T. A neurodegeneration-specific gene-expression signature of acutely isolated microglia from an amyotrophic lateral sclerosis mouse model. Cell Rep 2013;4(2):385-401.

[13] Christensson-Nylander I, Herrera-Marschitz M, Staines W, Hokfelt T, Terenius L, Ungerstedt U, Cuello C, Oertel WH, Goldstein M. Striato-nigral dynorphin and substance P pathways in the rat. I. Biochemical and immunohistochemical studies. Exp Brain Res 1986;64(1):169-192.

[14] Clark AK, Grist J, Al-Kashi A, Perretti M, Malcangio M. Spinal cathepsin S and fractalkine contribute to chronic pain in the collagen-induced arthritis model. Arthritis Rheum 2012;64(6):2038-2047.

[15] Clark AK, Yip PK, Grist J, Gentry C, Staniland AA, Marchand F, Dehvari M, Wotherspoon G, Winter J, Ullah J, Bevan S, Malcangio M. Inhibition of spinal microglial cathepsin S for the reversal of neuropathic pain. Proceedings of the National Academy of Sciences of the United States of America 2007;104(25):10655-10660.

[16] Cohen M, Matcovitch O, David E, Barnett-Itzhaki Z, Keren-Shaul H, Blecher-Gonen R, Jaitin DA, Sica A, Amit I, Schwartz M. Chronic exposure to TGFbeta1 regulates myeloid cell inflammatory response in an IRF7-dependent manner. EMBO J 2014;33(24):2906-2921.

[17] Denk F, Crow M, Didangelos A, Lopes DM, McMahon SB. Persistent Alterations in Microglial Enhancers in a Model of Chronic Pain. Cell Rep 2016;15(8):1771-1781.

[18] Dobin A, Davis CA, Schlesinger F, Drenkow J, Zaleski C, Jha S, Batut P, Chaisson M, Gingeras TR. STAR: ultrafast universal RNA-seq aligner. Bioinformatics 2013;29(1):15-21.

[19] Dunston CR, Griffiths HR, Lambert PA, Staddon S, Vernallis AB. Proteomic analysis of the anti-inflammatory action of minocycline. Proteomics 2011;11(1):42-51.

[20] Edwards RR, Wasan AD, Bingham CO, 3rd, Bathon J, Haythornthwaite JA, Smith MT, Page GG. Enhanced reactivity to pain in patients with rheumatoid arthritis. Arthritis Res Ther 2009;11(3):R61.

[21] Hashmi JA, Davis KD. Deconstructing sex differences in pain sensitivity. Pain 2014;155(1):10-13.

[22] Heiberg T, Finset A, Uhlig T, Kvien TK. Seven year changes in health status and priorities for improvement of health in patients with rheumatoid arthritis. Ann Rheum Dis 2005;64(2):191-195.

[23] Hua XY, Svensson CI, Matsui T, Fitzsimmons B, Yaksh TL, Webb M. Intrathecal minocycline attenuates peripheral inflammation-induced hyperalgesia by inhibiting p38 MAPK in spinal microglia. Eur J Neurosci 2005;22(10):2431-2440.

[24] Klein SL, Flanagan KL. Sex differences in immune responses. Nat Rev Immunol 2016;16(10):626-638.

[25] Kosek E, Altawil R, Kadetoff D, Finn A, Westman M, Le Maitre E, Andersson M, Jensen-Urstad M, Lampa J. Evidence of different mediators of central inflammation in dysfunctional and inflammatory pain--interleukin-8 in fibromyalgia and interleukin-1 beta in rheumatoid arthritis. J Neuroimmunol 2015;280:49-55.

[26] Lampa J, Westman M, Kadetoff D, Agreus AN, Le Maitre E, Gillis-Haegerstrand C, Andersson M, Khademi M, Corr M, Christianson CA, Delaney A, Yaksh TL, Kosek E, Svensson CI. Peripheral inflammatory disease associated with centrally activated IL-1 system in humans and mice. Proceedings of the National Academy of Sciences of the United States of America 2012;109(31):12728-12733.

[27] Leffler AS, Kosek E, Lerndal T, Nordmark B, Hansson P. Somatosensory perception and function of diffuse noxious inhibitory controls (DNIC) in patients suffering from rheumatoid arthritis. Eur J Pain 2002;6(2):161-176.

[28] Liao Y, Smyth GK, Shi W. featureCounts: an efficient general purpose program for assigning sequence reads to genomic features. Bioinformatics 2014;30(7):923-930.

[29] Lopes DM, Malek N, Edye M, Jager SB, McMurray S, McMahon SB, Denk F. Sex differences in peripheral not central immune responses to pain-inducing injury. 2017;7(1):16460.

[30] Love MI, Huber W, Anders S. Moderated estimation of fold change and dispersion for RNA-seq data with DESeq2. Genome Biol 2014;15(12):550.

[31] McInnes IB, Schett G. The pathogenesis of rheumatoid arthritis. N Engl J Med 2011;365(23):2205-2219.

[32] Meeus M, Vervisch S, De Clerck LS, Moorkens G, Hans G, Nijs J. Central sensitization in patients with rheumatoid arthritis: a systematic literature review. Semin Arthritis Rheum 2012;41(4):556-567.

[33] Mogil JS. Sex differences in pain and pain inhibition: multiple explanations of a controversial phenomenon. Nat Rev Neurosci 2012;13(12):859-866.

[34] Moller T, Bard F, Bhattacharya A, Biber K, Campbell B, Dale E, Eder C, Gan L, Garden GA, Hughes ZA, Pearse DD, Staal RG, Sayed FA, Wes PD, Boddeke HW. Critical data-based re-evaluation of minocycline as a putative specific microglia inhibitor. Glia 2016;64(10):1788-1794.

[35] Nieto FR, Clark AK, Grist J, Hathway GJ, Chapman V, Malcangio M. Neuron-immune mechanisms contribute to pain in early stages of arthritis. Journal of neuroinflammation 2016;13(1):96.

[36] Nikodemova M, Watters JJ. Efficient isolation of live microglia with preserved phenotypes from adult mouse brain. J Neuroinflammation 2012;9:147.

[37] Noristani HN, Gerber YN, Sabourin JC, Le Corre M, Lonjon N, Mestre-Frances N, Hirbec HE, Perrin FE. RNA-Seq Analysis of Microglia Reveals Time-Dependent Activation of Specific Genetic Programs following Spinal Cord Injury. Front Mol Neurosci 2017;10:90.

[38] Orazzo C, Pieribone VA, Ceccatelli S, Terenius L, Hokfelt T. CGRP-like immunoreactivity in A11 dopamine neurons projecting to the spinal cord and a note on CGRP-CCK cross-reactivity. Brain Res 1993;600(1):39-48.

[39] Picelli S, Bjorklund AK, Faridani OR, Sagasser S, Winberg G, Sandberg R. Smart-seq2 for sensitive full-length transcriptome profiling in single cells. Nature methods 2013;10(11):1096-1098.

[40] Picelli S, Faridani OR, Bjorklund AK, Winberg G, Sagasser S, Sandberg R. Full-length RNA-seq from single cells using Smart-seq2. Nature protocols 2014;9(1):171-181.

[41] Pollard LC, Ibrahim F, Choy EH, Scott DL. Pain thresholds in rheumatoid arthritis: the effect of tender point counts and disease duration. J Rheumatol 2012;39(1):28-31.

[42] Quadros AU, Pinto LG, Fonseca MM, Kusuda R, Cunha FQ, Cunha TM. Dynamic weight bearing is an efficient and predictable method for evaluation of arthritic nociception and its pathophysiological mechanisms in mice. Sci Rep 2015;5:14648.

[43] Racine M, Tousignant-Laflamme Y, Kloda LA, Dion D, Dupuis G, Choiniere M. A systematic literature review of 10 years of research on sex/gender and experimental pain perception - part 1: are there really differences between women and men? Pain 2012;153(3):602-618.

[44] Racine M, Tousignant-Laflamme Y, Kloda LA, Dion D, Dupuis G, Choiniere M. A systematic literature review of 10 years of research on sex/gender and pain perception - part 2: do biopsychosocial factors alter pain sensitivity differently in women and men? Pain 2012;153(3):619-635.

[45] Salter MW, Beggs S. Sublime microglia: expanding roles for the guardians of the CNS. Cell 2014;158(1):15-24.

[46] Schwarz JM, Sholar PW, Bilbo SD. Sex differences in microglial colonization of the developing rat brain. J Neurochem 2012;120(6):948-963.

[47] Shemer A, Erny D, Jung S, Prinz M. Microglia Plasticity During Health and Disease: An Immunological Perspective. Trends in immunology 2015;36(10):614-624.

[48] Sorge RE, Mapplebeck JC, Rosen S, Beggs S, Taves S, Alexander JK, Martin LJ, Austin JS, Sotocinal SG, Chen D, Yang M, Shi XQ, Huang H, Pillon NJ, Bilan PJ, Tu Y, Klip A, Ji RR, Zhang J, Salter MW, Mogil JS. Different immune cells mediate mechanical pain hypersensitivity in male and female mice. Nat Neurosci 2015;18(8):1081-1083.

[49] Su J, Gao T, Shi T, Xiang Q, Xu X, Wiesenfeld-Hallin Z, Hokfelt T, Svensson CI. Phenotypic changes in dorsal root ganglion and spinal cord in the collagen antibody-induced arthritis mouse model. J Comp Neurol 2015;523(10):1505-1528.

[50] Taves S, Berta T, Liu DL, Gan S, Chen G, Kim YH, Van de Ven T, Laufer S, Ji RR. Spinal inhibition of p38 MAP kinase reduces inflammatory and neuropathic pain in male but not female mice: Sex-dependent microglial signaling in the spinal cord. Brain Behav Immun 2016;55:70-81.

[51] Tay TL, Mai D, Dautzenberg J, Fernandez-Klett F, Lin G, Sagar, Datta M, Drougard A, Stempfl T, Ardura-Fabregat A, Staszewski O, Margineanu A, Sporbert A, Steinmetz LM, Pospisilik JA, Jung S, Priller J, Grun D, Ronneberger O, Prinz M. A new fate mapping system reveals context-dependent random or clonal expansion of microglia. Nat Neurosci 2017;20(6):793-803.

[52] ten Klooster PM, Veehof MM, Taal E, van Riel PL, van de Laar MA. Changes in priorities for improvement in patients with rheumatoid arthritis during 1 year of anti-tumour necrosis factor treatment. Ann Rheum Dis 2007;66(11):1485-1490.

[53] Theodorsson E, Rugarn O. Radioimmunoassay for rat galanin: immunochemical and chromatographic characterization of immunoreactivity in tissue extracts. Scand J Clin Lab Invest 2000;60(5):411-418.

[54] Trapnell C, Williams BA, Pertea G, Mortazavi A, Kwan G, van Baren MJ, Salzberg SL, Wold BJ, Pachter L. Transcript assembly and quantification by RNA-Seq reveals unannotated transcripts and isoform switching during cell differentiation. Nat Biotechnol 2010;28(5):511-515.

[55] Tsuda M, Beggs S, Salter MW, Inoue K. Microglia and intractable chronic pain. Glia 2013;61(1):55-61.

[56] Tsuda M, Shigemoto-Mogami Y, Koizumi S, Mizokoshi A, Kohsaka S, Salter MW, Inoue K. P2X4 receptors induced in spinal microglia gate tactile allodynia after nerve injury. Nature 2003;424(6950):778-783.

[57] Wang L, Wang S, Li W. RSeQC: quality control of RNA-seq experiments. Bioinformatics 2012;28(16):2184-2185.

[58] Vawter MP, Evans S, Choudary P, Tomita H, Meador-Woodruff J, Molnar M, Li J, Lopez JF, Myers R, Cox D, Watson SJ, Akil H, Jones EG, Bunney WE. Gender-specific gene expression in post-mortem human brain: localization to sex chromosomes. Neuropsychopharmacology 2004;29(2):373-384.

**Figure legends**

**Figure 1:** **CAIA induces transient joint inflammation and persistent mechanical hypersensitivity in male and female mice.** **(A)** Joint inflammation was assessed visually by counting the number of inflamed digits, paws, wrists and ankles (arthritis score). Male and female CAIA groups developed transient joint inflammation while saline injected controls did not show any signs of inflammation. **(B)** Mechanical hypersensitivity was assessed by measuring paw withdrawal thresholds using von Frey filaments. Both male and female CAIA groups developed persistent mechanical hypersensitivity while the saline injected groups did not. Data is presented as mean ± SEM, males n=8; females n=12 mice per group, CAIA vs. saline, ***p>0.001, **p>0.01, *p>0.05.

**Figure 2: CAIA does not alter the expression levels of CGRP, SP or galanin in the spinal dorsal horn.** Relative expression based on assessment of fluorescence intensity of **(A)** CGRP, **(B)** SP and **(C)** galanin, as well as **(D)** representative images of immunoreactivity in the lumbar spinal cord of male and female mice 15 and 54 days after CAIA induction. Data is presented as mean ± SEM, n=8 mice per group, male vs. female CAIA mice on day 54.

**Figure 3: CAIA leads to an increase in spinal GFAP and IBA-1 immunoreactivity in male and female mice.** Relative expression based on assessment of fluorescence intensity of **(A)** GFAP and **(C)** IBA-1 and **(B,D)** representative images of immunoreactivity in the lumbar spinal cord of male and female mice 15 and 54 days after CAIA induction. Significant increase of the astrocyte marker GFAP was observed only in the late phase of CAIA in both males and females, while increase in the signal intensity of the microglia marker IBA-1 was detected in both CAIA phases in both male and female mice. Data is presented as mean ± SEM, n=8 mice per group, CAIA vs. saline, ***p>0.001, **p>0.01.

**Figure 4: Intrathecal minocycline and pentoxifylline treatment reverses late-stage mechanical hypersensitivity in the CAIA model in male mice only.** Bar graphs showing the effects of spinal microglia inhibition on withdrawal thresholds of mice. Both minocycline and pentoxyfilline reversed mechanical hypersensitivity in males **(B,D)** but not females **(A,C)** 6 hours after intrathecal administration. Data is presented as mean ± SEM, n=6 per group; one-way ANOVA, CAIA+vehicle vs. CAIA+inhibitor, **p>0.01, *p>0.05.

**Figure 5: Microglia from whole naïve spinal cord do not show prominent sex differences in number or expression level. (A)** Gating strategy employed to sort CD11b positive live single immune cells. Shown are serial dot plots of the events recorded by the flow cytometer, with gates indicating chosen populations that are then displayed in the next adjacent plot. Sequentially: Side scatter (SSC) vs Forward scatter (FSC) to distinguish leukocytes from debris using their complexity and size; Forward scatter width (FSC-W) vs area (FSC) to eliminate leukocyte doublets; FSC vs the live-dead cell marker Sytox blue to eliminate dead cells; and finally, FSC versus CD45, a marker for all immune cells. Only the events contained in this final CD45+ gate are taken forward for analysis of CD11b positivity. **(B)** No differences could be detected in the number of live microglia obtained from whole spinal cord of male and female mice. **(C)** Myeloid cell markers Iba-1 and CD11b did not show differential expression, while XY linked genes Xist, Eif2s3y and Ddx3y were significantly more highly expressed in female or male mice, respectively.

**Figure 6: Macrophage infiltration into the lumbar dorsal horn is absent or negligible.** Dot plots of myeloid cells (gated on live CD45+ positive singlets) from naïve and CAIA animals isolated during the inflammatory phase (day 7 and day 19). Immune cells were stained for CD11b and Fcrls, a microglial cell marker not present in infiltrating macrophages. Both naïve and CAIA groups have a similar proportion of CD11b^+^ /Fcrls^+^ cells (over 99%) indicating that macrophage infiltration during the inflammatory phase of CAIA is negligible. The rightmost panel displays a fluorescence minus one (FMO) control, where the Fcrls primary antibody was omitted from the staining panel.

**Figure 7: Flow cytometry analysis points to fewer dorsal horn microglial cells in females than males.** Representative gating dot plots of CD45^+^ CD11b^+^ microglial cells from saline and CAIA **(A)** female and **(B)** male mice during the post-inflammatory phase (days 54 to day 60). **(C)** Percentage of CD45^+^ CD11b^+^ microglia amongst CD45^+^ immune cells. Females show a significant decrease in lumbar dorsal horn microglial and CD45^+^ cell numbers as compared to males (*p<0.05, one-way ANOVA) whereas the number of live cells does not differ (see Suppl. Fig. S1, available at http://links.lww.com/PAIN/A666). No differences were found between the saline and CAIA groups. Data is presented as mean ± SEM.

**Figure 8. Principal component analysis reveals marked stratification between male and female microglia that are mostly due to X-Y linked genetic differences. (A)** PCA of male and female microglia samples from saline and post-inflammatory CAIA groups. **(B)** PCA of male and female microglia samples in which significantly differentially expressed X and Y linked genes have been excluded from the analysis. **(C)** Volcano plot depicting differentially expressed genes between male and female microglia obtained using the Deseq2 algorithm. Colored dots represent significantly dysregulated genes at adjusted p<0.05, logFC>1 (dark blue for males, dark pink for females) and at adjusted p<0.05, logFC<1 (light blue for males, light pink for females).

**Table 1: Primers used for qRT-PCR of FACS-purified mouse spinal cord microglia.** All primers were tested for their efficiency and specificity (via melt curve and agarose gel analysis).
